# Diastasis Recti Abdominis Rehabilitation in the Postpartum Period: A Scoping Review of Current Clinical Practice

**DOI:** 10.1007/s00192-024-05727-1

**Published:** 2024-02-10

**Authors:** Anastasia Skoura, Evdokia Billis, Dimitra Tania Papanikolaou, Sofia Xergia, Charis Tsarbou, Maria Tsekoura, Eleni Kortianou, Ioannis Maroulis

**Affiliations:** 1 Laboratory of Clinical Physiotherapy and Research, Department of Physiotherapy, School of Health Rehabilitation Sciences, University of Patras, Building B, Central Campus 26504 Rio, Patras, Achaia Greece; 2 Department of Physiotherapy, School of Health Sciences, University of Thessaly, Lamia, Greece; 3 Faculty of Medicine, School of Health Sciences, University of Patras, Rio Patras, Greece

**Keywords:** Diastasis recti abdominis, Postpartum, Rehabilitation

## Abstract

**Introduction and hypothesis:**

Despite exercise being the standard approach to diastasis recti abdominis (DRA) rehabilitation, there is no consensus on the most effective exercise routine and adjunct modalities for reducing DRA and improving functional parameters. The present study is aimed at investigating evidence for DRA rehabilitation in postpartum women, as well as knowledge gaps and areas for future research.

**Methods:**

For this scoping review a systematic search was conducted in MEDLINE, AMED, CINAHL, Embase, ScienceDirect, Scopus, and PEDro up to November 2022. Selection criteria included studies investigating exercise therapy interventions both with and without adjunct modalities for postpartum DRA. Sample characteristics, diagnostic criteria, program design, and outcome measures were recorded. Critical appraisal of clinical trials was performed using PEDro classification.

**Results:**

Twenty-eight studies were included: 14 clinical trials, 3 case series, and 11 observational studies. DRA exercises that focused on deep and superficial muscles, pelvic floor muscles, respiratory maneuvers, functional exercises, or alternative interventions (yoga, suspension training, hypopressive exercise) and adjunct modalities showed promising results in reducing the inter-recti distance and related dysfunction. However, there was great variability in diagnostic criteria and methods, DRA severity, time post-birth, and exercise program design.

**Conclusions:**

Reviewed studies provide valuable insights into exercise therapy, but it is important to recognize their limitations, as variability in diagnostic criteria, sample characteristics, and exercise program design hinder the generalizability of the findings. Further high-quality research is needed to strengthen the evidence in this area and provide reliable recommendations for clinical practice.

**Supplementary information:**

The online version contains supplementary material available at 10.1007/s00192-024-05727-1.

## Introduction

Diastasis recti abdominis (DRA) is a connective tissue condition characterized by a separation of the two rectus abdominis (RA) muscles along the linea alba (LA) due to stretching and thinning. Separation is visible during an abdominal contraction, appearing either like a bulge or invagination through the midline of the anterior abdominal wall [1]. DRA primarily affects women antenatally and postpartum; prevalence varies between 66 and 100% in the final trimester of pregnancy and up to 53% within 24 h of delivery [2, 3]. Natural resolution is reported during the first 8 weeks postpartum, after which time spontaneous resolution plateaus [2, 4, 5]. However, some researchers suggest that recovery might still be ongoing at 6 months postpartum [6], and, despite limited research, DRA is found to be present in 36% of women at 12 months postnatally [2, 3, 7].

Assessment and diagnosis of DRA are performed via inter-recti distance (IRD) [5] measurement. Ultrasound and calipers are recommended as the most reliable IRD assessment methods [8, 9]. Currently, there is no consensus about cut-off points for diagnosis, although some researchers consider ultrasound measurements of 2.2–2.3 cm at the umbilicus level to be clinically important [5, 10]. Beer et al. [1], utilizing healthy nulliparous women, classified ultrasound-measured IRDs during rest with DRA if >1.5 cm at the xiphoid, >2.2 cm at 3 cm above the umbilicus, and >1.6 cm at 2 cm below the umbilicus.

The abdominal wall plays a significant role in maintaining posture, trunk and pelvic mobility and stability, adequate breathing quality, and abdominal viscera support. Stretching and thinning of the LA during gestation is a result of connective tissue laxity and mechanical strain from hormonal changes and greater intra-abdominal pressure [2, 11]. Abdominal bulging results from visceral protrusion between rectus bellies, whereas abdominal invagination results from linea alba posterior distortion. Both clinical signs, evident in DRA, can cause significant aesthetic concerns for postpartum women. Additionally, modifications in muscle pull angles may alter body mechanics and impair the ability of the abdominal muscle to generate force and the ability of the fascia to transfer loads across the midline [12]. Although there is weak evidence on the effects of DRA on trunk and pelvic dysfunctions or imbalances, some researchers claim that DRA-associated muscle changes may impair abdominal muscle strength, modify breathing patterns, and lead to low back or pelvic girdle pain, or other pelvic floor disorders [10, 13–16].

According to the latest guidelines, DRA management should be primarily conservative, and physiotherapy is the gold standard approach [17]. Surgical intervention, involving the reduction of the IRD through plication of the linea alba and anterior rectus sheath with or without a mesh, is typically reserved for severe cases where conservative treatment fails, no further reduction is achieved, or a concomitant symptomatic hernia is present [17, 18]. However, owing to surgical complications and the potential recurrence of DRA with subsequent pregnancies, a conservative approach is generally recommended for at least 6 months [17]. However, although rehabilitation focusing on various exercises, including pelvic floor muscle (PFM) exercises, transversus abdominis (TrA) exercises, hypopressive abdominal training [19–22], etc., is promising [21, 23], most studies are of low methodological quality [24] and present great heterogeneity regarding DRA severity, IRD measurement methods, cut-off points, etc., thus indicating no consensus on a standardized rehabilitation protocol [24, 25]. Given the above limitations, a scoping review was conducted to systematically map research on therapeutic exercise and adjunct modalities for DRA postpartum, with the objective of qualitatively synthesizing their findings and comparing their designs. The scoping review was aimed at addressing the following research questions:

1. What are the current exercise interventions and adjunct modalities utilized for DRA rehabilitation in postpartum women?
2. What is the impact of exercise therapy and adjunct modalities on IRD reduction and related functional outcomes in postpartum women with DRA?
3. What are the existing knowledge gaps and limitations in the current literature on DRA rehabilitation practices for postpartum women?

## Materials and Methods

This scoping review was designed according to the Joanna Briggs Institute Updated Guidelines of 2020 [26] and also follows the Preferred Reporting Items for Systematic Reviews and Meta-Analyses–Scoping Review (PRISMA-ScR) reporting guidelines [27, 28] (http://www.prisma-statement.org/Extensions/ScopingReviews). The study protocol was registered with OSF-Standard Pre-Data Collection Registration on Open Science Framework (OSF) (https://osf.io/hm2t4/?view_only=6f11dfcdf0ee4e42852081a6e49f9616). An electronic search was conducted between March and November 2022 on databases such as MEDLINE, CINAHL, Embase, ScienceDirect, Scopus, and PEDro, for studies that satisfied the inclusion criteria. Keywords were based on the Population, Concept, and Context (PCC) framework and included the terms "DRA" or "diastasis recti" or "rectus abdominis diastasis" or "recti divarication" and "postpartum women" or "parous women" and "physiotherapy" or "physical therapy" or "exercise" or "training" or "rehabilitation." Boolean logic (AND, OR, and NOT) was employed to generate combinations of search strings. The final search strategy for MEDLINE can be found in Appendix 1.

Eligibility criteria included experimental studies (randomized controlled trials [RCTs], controlled clinical trials [CCTs], case series) or observational/descriptive studies (cohorts, case–control, cross-sectional, longitudinal, prospective studies) containing the above terms, articles published in English and in full-text, without any limitations regarding publication date. Exclusion criteria were single-case studies, reviews, and clinical commentaries, studies in which none of the study groups entailed exercise interventions, observational studies restricted solely to healthy nulliparous subjects, and studies proposing finger-widths or tape measures as IRD outcomes. Results were scanned manually, and articles not complying with the above criteria were excluded.

To increase consistency, all three reviewers participating in the study selection and data extraction studied the PRISMA-ScR guidelines, agreed to the aforementioned screening and data extraction method, and reviewed the articles. Disagreements regarding study selection and data extraction were resolved by consensus and subsequent discussion amongst reviewers. Two reviewers developed a data-charting form and independently charted data, discussed results, and modified data-charting accordingly. Data were charted according to study design, and sample characteristics, interventions, and outcomes were tabulated. Summarized data are presented in Appendix 2. Data were interpreted and summarized by the three reviewers.

Critical appraisal of RCTs was attained by PEDro classification for clinical trials. The two assigned reviewers independently assessed and scored each scale’s item, considering that total PEDro scores of 0–3 are considered “poor,” 4–5 “fair,” 6–8 “good,” and 9–10 “excellent.” Disagreements between the reviewers were settled with the help of a third reviewer when necessary.

This scoping review was exempt from institutional review board approval as it did not involve primary data collection or human subjects. This study is part of a broader research project approved by the Research Ethics Committee of the University of Patras, with internal code 16192.

## Results

**Fig. 1:**
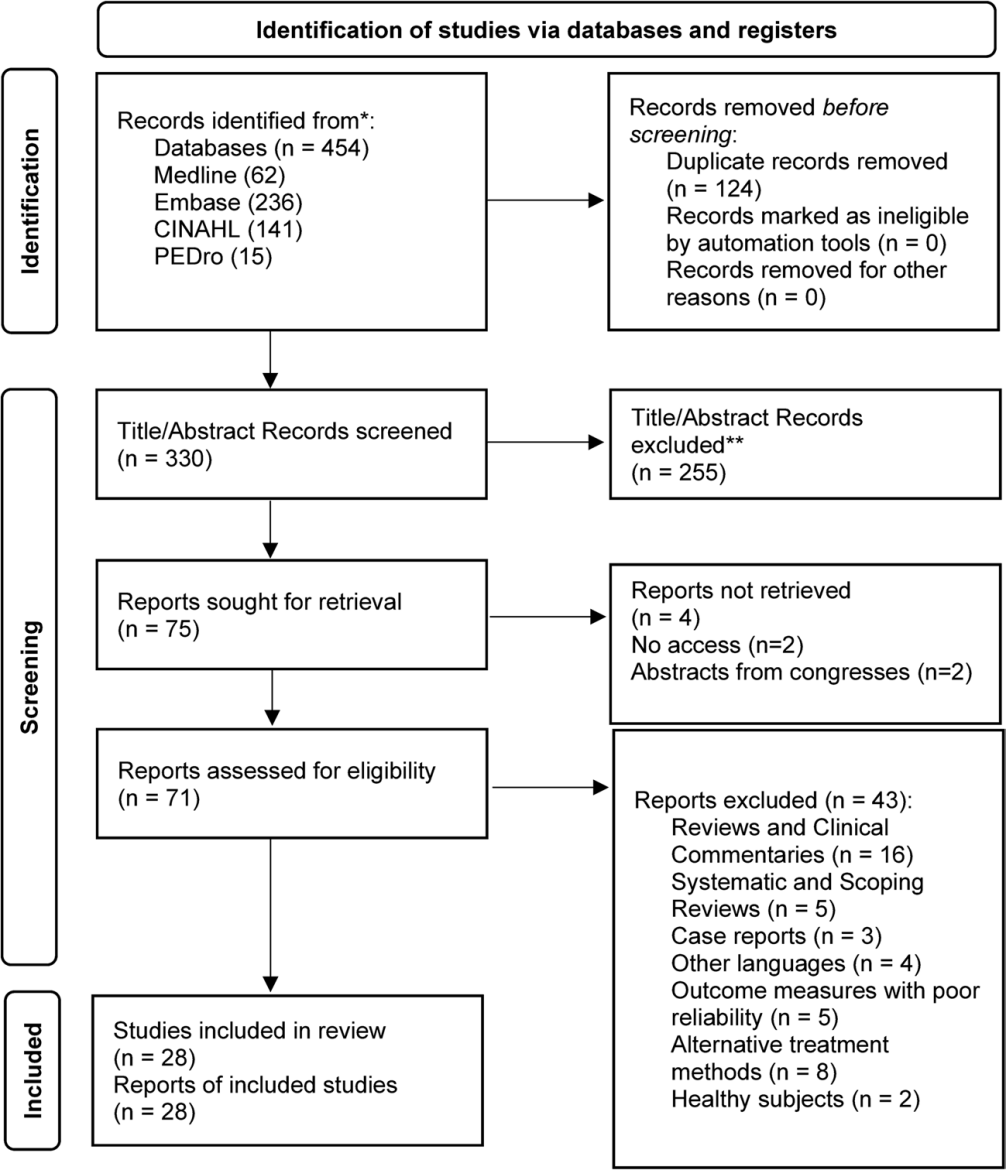
Flow diagram demonstrating the scoping review of literature for diastasis recti abdominis rehabilitation according to Preferred Reporting Items for Systematic Reviews and Meta-Analyses guidelines [28]

The database search identified 454 records. After removing duplicates, 330 articles were eligible for screening. Title and abstract screening removed another 255 records, leaving 75 articles for full-text review. Four reports were unavailable in full-text, and of the remaining 71, a total of 43 were excluded (Fig. 1). Finally, 28 full-text articles met the inclusion criteria: 14 clinical trials (13 RCTs, 1 CCT), 3 case series, and 11 observational/descriptive studies (cohorts, case–control, cross-sectional, longitudinal, and prospective studies).

**Fig. 2:**
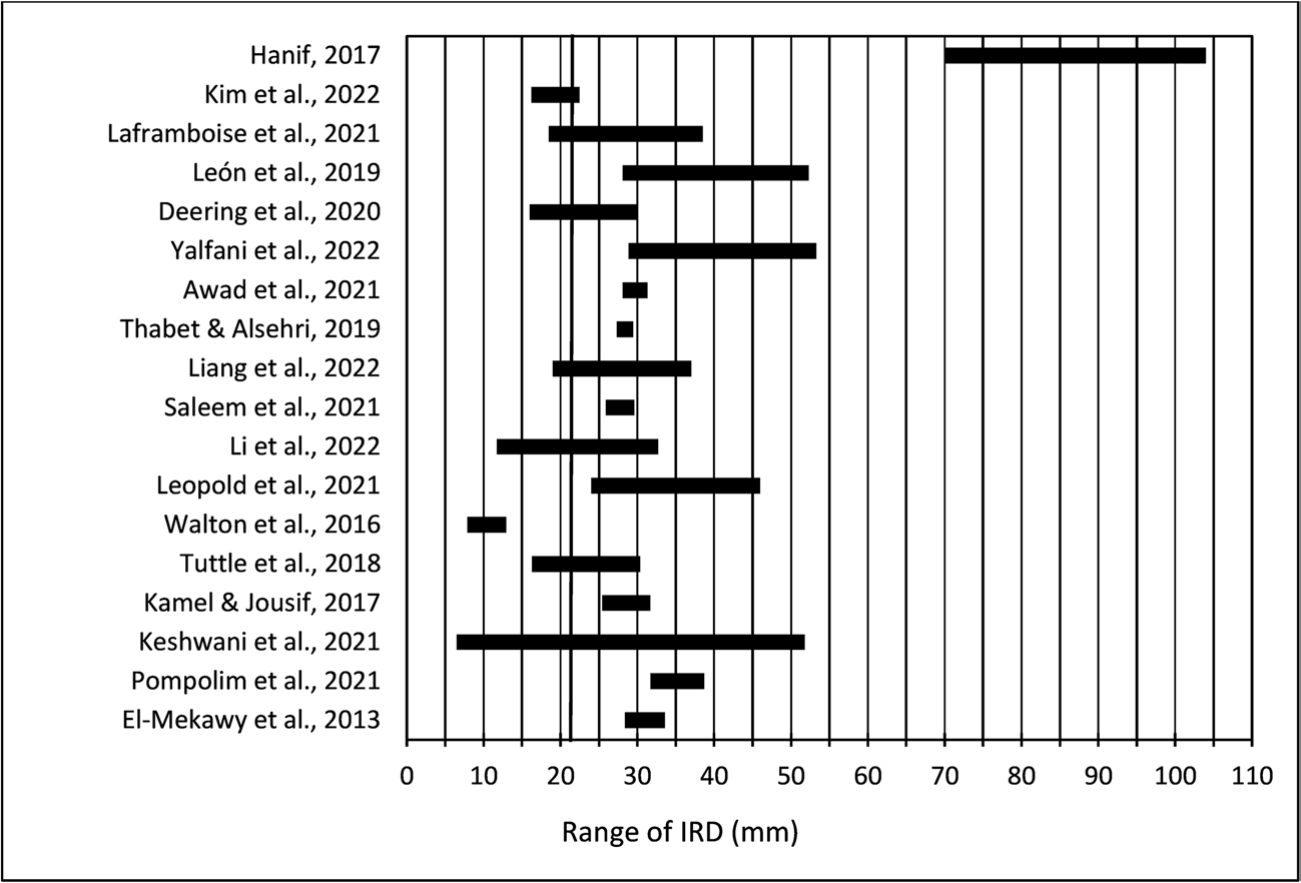
Range of inter-recti distance (*IRD*; mm) found within the participant population of each study. Notably, there is substantial variability across studies, with some including women with severe diastasis recti abdominis (*DRA*; IRD >50 mm) and others including women with smaller IRDs, which could be categorized as nonpathological or borderline DRA (6-22 mm). Certain studies featured a more diverse IRD range (6-50 mm), also encompassing women with moderate DRA. The bold, vertical line in the figure denotes the threshold for classifying DRA according to the criteria established by Beer et al. [1], with an IRD greater than 22 mm considered indicative of DRA

The reviewed studies had varying sample sizes (3–129) and used different cut-off points for DRA diagnosis, such as 2.0 cm [29–32], 2.2 cm [12], or 2.5 cm [33–37] above the umbilicus during rest, and 1.5–3.0 cm above the umbilicus during a head-lift or curl-up [38–40]. Three studies proposed cut-offs of two finger-widths at the umbilicus [21] or anywhere in the midline [23, 41] during a head-lift and subsequently assessed with ultrasound, whereas 12 studies did not use any cut-off points [22, 37, 42–51]. Additionally, IRDs varied broadly across studies (Fig. 2). Although most samples had ultrasound-measured [12, 21–23, 30–34, 36, 37, 41, 43, 46–51] or caliper-measured [29, 35, 38–40, 42–45, 52] IRDs between 2.5 and 5 cm, two studies [43, 47] encompassed IRDs <2.5 cm, whereas one case series [52] included women with IRDs >7 cm. However, 7 studies [21, 23, 32, 34, 37, 45, 48] included postpartum women with combined normal and borderline IRD, according to established classification systems [1].

In clinical trials, the IRD was assessed under various conditions, including rest [23, 33, 37, 47, 48], head-lift [35, 38, 52], curl-up maneuver [29, 39, 40, 42–44], or combinations [21, 45, 46]. Observational studies also utilized combinations of rest and head-lift/curl-up maneuvers [30, 32, 49] with additional conditions, such as TrA [12, 50, 51] and PFM contractions [31, 36], sit-ups [38], leg raise or side planks [41], and abdominal hypopressive exercises (AHEs) [22]. One study [34] provided no information about the position/condition of patients during the assessment.

**Fig. 3:**
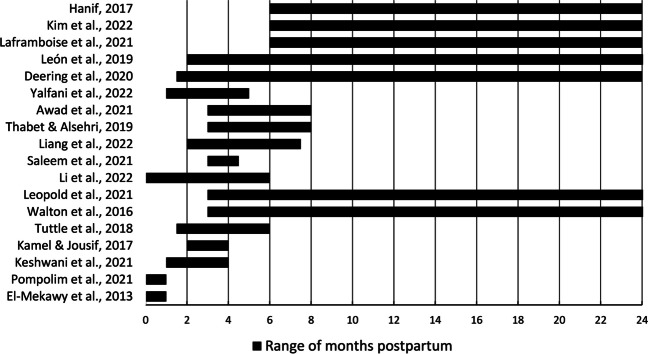
Range of time postpartum per study (months) for participants in each study, at the time of participant enrollment, indicating the initiation of rehabilitation. Interestingly, most of the studies included women who were within the first 6 months post-birth, a period during which a spontaneous reduction of the inter-recti distance may overlap with the intervention effect. Only three studies exclusively enrolled women who were at least 6 months postpartum, whereas for some studies the maximum time post-birth was 3 years

Time postpartum varied broadly (Fig. 3), including women who were between 6 h and 6 months postpartum [21, 23, 29, 33, 39, 40, 42, 48], less than 2 days postpartum [40, 42], less than 4 months postpartum [23, 33], between 1 week and 6 months postpartum [21, 29, 39, 48], between 2 and 8 months postpartum [34, 44, 46], over 6 months postpartum [45, 47, 52], whereas two studies [32, 43] enrolled women at 3 months to 3 years postpartum.

Exercise program duration varied in most studies between 6 and 12 weeks, except for one study [40], which applied two exercise sessions during the first 24 h postpartum (Appendix 2). Exercise frequency ranged from once [34, 35, 37, 48], twice [22, 47], or three times weekly [29, 33, 39, 42–46, 52]. Twelve studies utilized home exercise programs; 8 recommending daily exercise sessions [23, 32–35, 37, 42, 44], 2 [22, 43] recommending two or three times per week, and 2 [46, 47] recommending regular exercise (as often as possible), without additional specifications. Six studies [21, 29, 39, 45, 48, 52] provided no information about home exercises. Exercise progression was mentioned in 13 studies, including progression in repetitions and sets [33, 34, 40, 43, 46], exercise complexity [23, 37, 39, 45, 48], multi-parameter progression [32, 47], and 1 study [32] reported modifications according to patients’ ability and performance, without providing exercise progression details.

### Quality of Included Studies

Among 14 RCTs/CCTs (Table 1), 9 were rated as good quality [21, 23, 33, 34, 39, 44–47], 4 as fair [29, 42, 43, 48], and 1 as poor [40], scoring 6–8 points, 4–5 points, and 3 points on the PEDro scale respectively. All RCTs/CCTs stated their purpose clearly, adequately described eligibility/selection criteria, and provided appropriate outcomes. Participant samples varied and in all but 2 RCTs [34, 46] sample size calculations were not provided. Case series and observational studies were charted separately.

**Table 1:** PEDro classification score for the randomized controlled trials included

Study	Eligibility criteria	Random allocation	Concealed allocation	Baseline comparability	Subject blinding	Therapist blinding	Assessor blinding	Adequate follow-up	Intention-to-treat analysis	Between- group statistical comparisons	Point measures and variability data	PEDro score
El-Mekawy et al. [42]	✓	✓						✓		✓	✓	4/10
Walton et al. [43]	✓	✓					✓	✓		✓	✓	5/10
Kamel & Yousif [33]	✓	✓		✓			✓	✓		✓	✓	6/10
Awad et al. [46]	✓	✓	✓	✓				✓		✓	✓	6/10
Tuttle et al. [21]	✓	✓	✓	✓			✓			✓	✓	6/10
Thabet & Alshehri [44]	✓	✓		✓			✓	✓		✓	✓	6/10
Keshwani et al. [23]	✓	✓		✓			✓	✓		✓	✓	6/10
Laframboise et al. [45]	✓	✓	✓	✓				✓		✓	✓	6/10
Saleem et al. [29]	✓	✓	✓	✓						✓	✓	5/10
Pompolim et al. [40]	✓							✓		✓	✓	3/10
Liang et al. [34]	✓	✓	✓	✓			✓	✓	✓	✓	✓	8/10
Kim et al. [47]	✓	✓		✓				✓	✓	✓	✓	6/10
Yalfani et al. [39]	✓	✓	✓	✓			✓			✓	✓	6/10
Li et al. [48]	✓	✓		✓				✓		✓	✓	5/10

### Contribution of Individual Exercises On IRD Reduction

#### Abdominal Muscle Training

Abdominal muscle training was performed in 16 studies, with 10 utilizing exercises for RA muscles (crunches/curl-ups [29, 39, 43, 47], sit-ups [33, 34], and posterior pelvic tilts [33, 34, 42–44, 47]), and 7 [33–35, 40, 42–44] including exercises for oblique abdominals (trunk/Russian twists, twisted curl-ups, etc.; Appendix 2). Six studies included eccentric contractions [23, 33, 34, 43, 44, 47] (i.e., reverse sit-ups and reverse trunk twists). Eleven studies [21–23, 32, 33, 37, 39, 40, 42, 44, 47] included exercises activating TrA muscles, such as abdominal drawing-in maneuvers/static abdominal contractions. Exercise progressions included a combination of abdominal muscle control and distal extremity movements [23, 29, 32, 39, 45, 46].

#### Rectus Abdominis Training

**Fig. 4:**
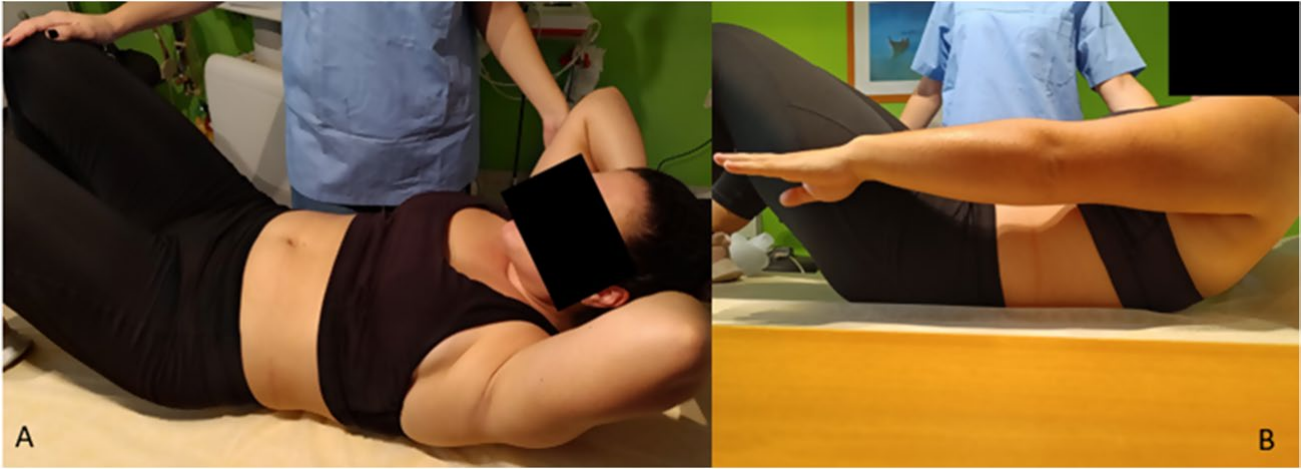
Abdominal muscle exercises (rectus abdominis focused) **A**. Crunch exercise. The patient is positioned in supine with legs bent and arms supporting the head. Then they are asked to dynamically elevate their head and upper torso until their shoulder blades lift off the surface. **B**. Curl-up exercise. Positioned in supine with bent legs and arms extended beside the hips, parallel to the bed, the patient is instructed to curl their upper back and shoulders in a controlled way, until their shoulder blades are off the bed while maintaining their lower back in contact with the bed. The arms remain parallel to the ground, reaching towards the feet

Several studies (observational [22, 30, 41, 50] and case–controls [49]) observed an immediate DRA closure (above and below the umbilicus) during abdominal crunch/curl-up maneuvers [30, 51] (Fig. 4). A fair-quality RCT [43] reported statistically significant improvements in IRD closure with “traditional” curl-ups compared with plank exercises (Table 2). Another fair-quality RCT [29] found abdominal crunches to be more effective than a double straight leg raise exercise protocol for reducing IRD. In an observational study [36], a head-lift maneuver seemed to have a similar effect to a twisted curl-up exercise on IRD reduction. Djivoh and De Jaeger [38], however, observed a significant IRD decrease during curl-ups and sit-ups compared with head-lifts. DRA reduction during curl-up in women with vaginal deliveries and in those with cesarean sections was found to be similar [50].

**Table 2:** Primary and secondary outcome measures per randomized controlled trial (*RCT*)

RCTs	Comparison between groups	Program duration	Between-groups differences in IRD reduction	Abdominal muscle function	Other outcomes
El-Mekawy et al. [42]	Abdominal binding vs abdominal binding + abdominal exercise	6 weeks	In favor of abdominal binding + abdominal exercise group	In favor of abdominal binding + abdominal exercise group	Waist/hip ratio decrease in favor of abdominal binding + abdominal exercise
Walton et al. [43]	Abdominal exercises + plank vs abdominal exercises + crunch + binding	6 weeks	NS*	–	NS differences for PFDI and ODI scores
Kamel & Yousif [33]	Abdominal exercises + NMES vs abdominal exercises + abdominal binding	8 weeks	In favor of abdominal exercises + NMES group	In favor of abdominal exercises + NMES group (peak torque)	–
Tuttle et al. [21]	TrA exercise vs tape vs TrA exercise + tape vs control	12 weeks	NS**	–	NS differences for PFDI-20 and RMDQ scores
Thabet & Alshehri [44]	Core stability + “traditional” exercises + abdominal binding vs “traditional” exercises	8 weeks	In favor of core stability + “traditional” exercises + abdominal binding group	–	PF-10 improvement in favor of the core stability + “traditional” exercises + abdominal binding group
Keshwani et al. [23]	Abdominal exercise vs abdominal binding vs abdominal exercise + abdominal binding vs control	12 weeks	NS***	NS*****	NS****** for PFDI, IFSAC, body image
Laframboise et al. [45]	Online abdominal exercises vs control	12 weeks	In favor of online abdominal exercises group	NS	NS for weight
Pompolim et al. [40]	Stabilization exercises vs control	18 hours	In favor of stabilization exercises group above the umbilicus	–	–
Saleem et al. [29]	Stabilization exercises + crunches vs stabilization exercise + double straight leg raise	6 weeks	In favor of stabilization exercises + crunches group	–	ODI in favor of the stabilization exercises + crunches group
Awad et al. [46]	Progressive prone plank exercise + abdominal binding + advice vs abdominal binding + advice	8 weeks	In favor of progressive plank exercise + abdominal binding + advice group	–	–
Kim et al. [47]	Online exercise program vs offline exercise program	6 weeks	NS	Left RA thickness in favor of the offline group	Spouse category of MAPP-QOL in favor of the online group
Liang et al. [34]	Abdominal exercises + BAPFMT + NMES vs abdominal exercises + NMES	6 weeks	In favor of the abdominal exercises + BAPFMT + NMES group	–	SF-36 physical components summary in favor of the abdominal exercises + BAPFMT + NMES group
Yalfani et al. [39]	Suspension training vs isometric–isotonic stabilization exercise vs control	8 weeks	NS****	–	NS**** for lumbopelvic proprioception and control, postural static and dynamic instability, low back pain, and ODI score
Li et al. [48]	Yoga vs control	12 weeks	In favor of the yoga group	–	–

*IRD* inter-recti distance, *NS* nonsignificant, *NMES* neuromuscular electrical stimulation, *TrA* transversus abdominis, *MAPP-QOL* Maternal Quality Of Life Questionnaire, *BAPFMT* biofeedback-assisted pelvic floor muscle training, *PFDI* Pelvic Floor Disability Index, *ODI* Oswestry Disability Index, *RMDQ* Roland Morris Disability Questionnaire, *IFSAC* Inventory of Functional Status After Childbirth

*Greater reduction for abdominal exercises + crunch + binding group

**Significantly greater reduction in IRD at rest and during head lift in the groups with TRA exercise compared with control/tape

***Slightly smaller reduction in the abdominal exercise group compared with control who presented natural recovery

****Nonsignificant between the intervention groups. Significant differences in favor of the intervention vs control

*****Positive effects in strength (Cohen`s d(d)=0.5–0.7) in the exercise and combination groups

******Positive effects in body image (d=0.2–0.5) in the abdominal binding alone and combination groups

#### Transversus Abdominis Training

**Fig. 5:**
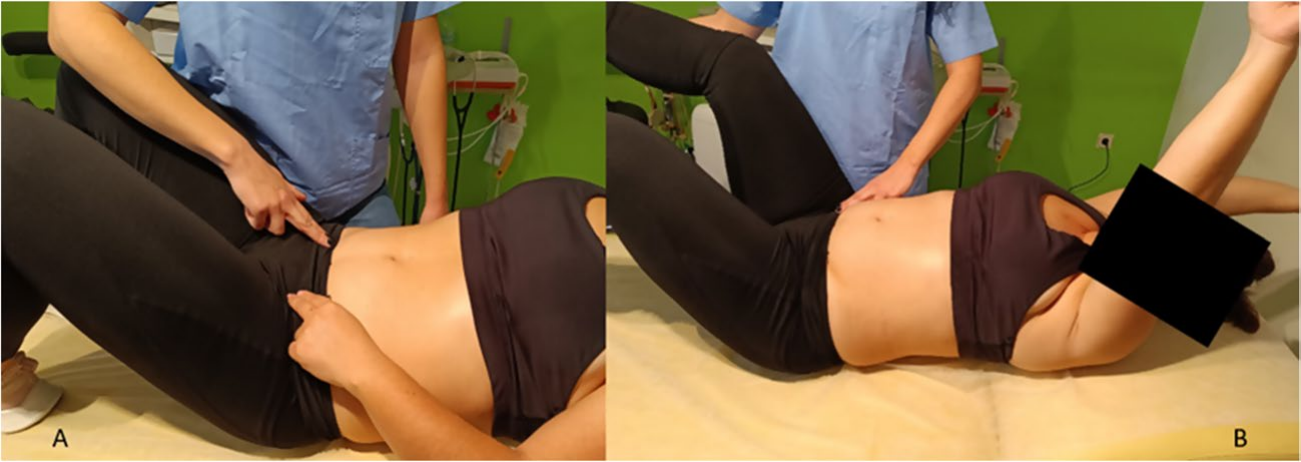
Activation of the transversus abdominis (TrA) muscle. **A**. The patient is instructed to take a deep breath keeping the abdomen relaxed and then to fully exhale slowly. At the end of expiration, the patient is asked to perform an abdominal drawing-in maneuver (ADIM); to pull the belly button towards the spine and upwards towards the thorax. The ADIM is considered to activate the deeper abdominal muscles (transversus abdominis and internal oblique), thus stabilizing the trunk. The physiotherapist can palpate deeper abdominal muscle activation on the lower abdominal wall, just medially of the anterior superior iliac spine. As the patient gains better control of the deeper abdominal muscles, they can be instructed to self-palpate muscle activation using two fingers. They are encouraged to sustain the contraction while breathing and audibly counting up to 10 (10 seconds). In clinical practice, transversus abdominis (TrA) activation is commonly integrated with pelvic floor muscle activation, as these muscle groups demonstrate a close collaborative relationship. B. TrA activation with distal extremity movement is a progression of the TrA activation exercise. After achieving a stable TrA contraction for at least 10 seconds while breathing, the exercise can be advanced by introducing movement in either the upper or lower extremities. As the patient gains sufficient control, both upper and lower extremities can be moved simultaneously, either in a parallel or crossed manner

**Fig. 6:**
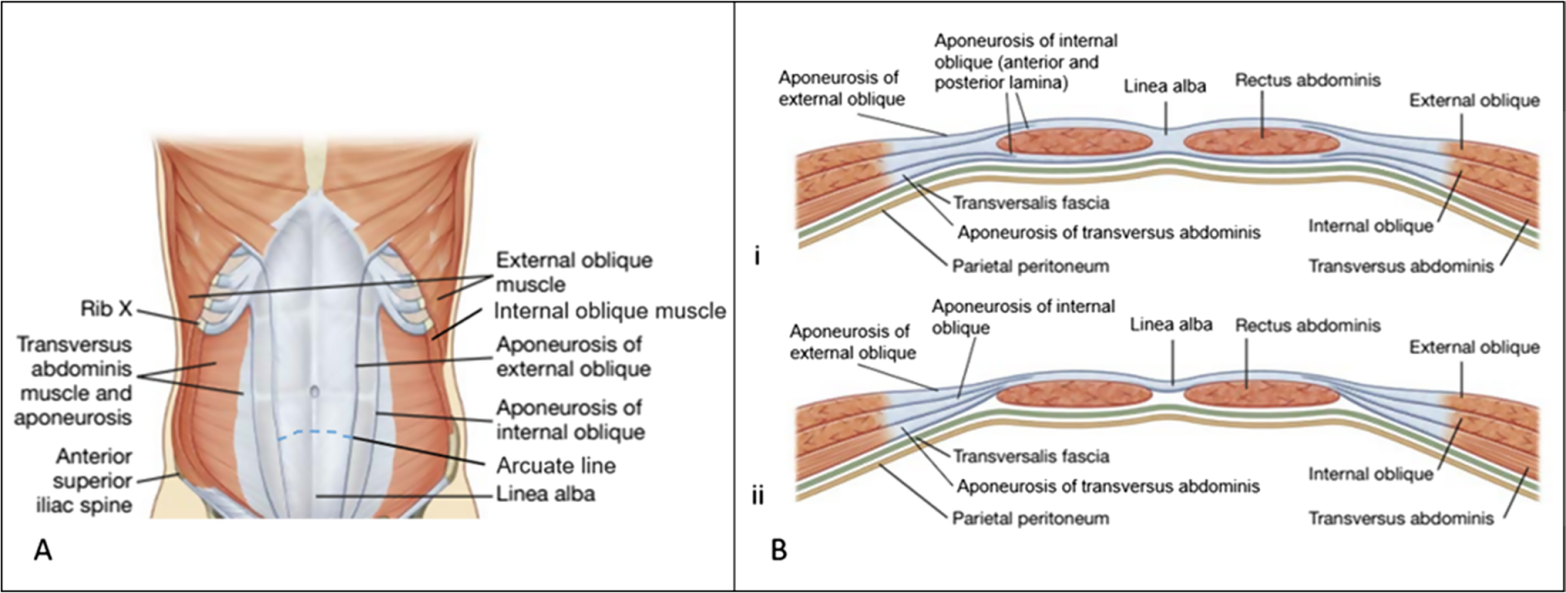
Anatomy of the abdominal wall. **A**. Anatomy of the abdominal wall (external oblique, internal oblique, linea alba and rectus sheath formed by the aponeuroses of the oblique and transversus abdominis muscles). Rectus abdominis muscle is also illustrated within the rectus sheath. **B**. cross-section of the abdominal muscles on i) upper three-quarters of the rectus sheath (above the arcuate line) where the anterior rectus sheath is formed by the aponeurosis of the external oblique and the anterior lamina of the aponeurosis of the internal oblique, while the posterior rectus sheath is formed by the posterior lamina of the aponeurosis of the internal oblique and the aponeurosis of the transversus abdominis muscle ii) lower quadrant of the rectus sheath (below the arcuate line) where the anterior rectus sheath is formed by the aponeurosis of the external oblique, the anterior and posterior lamina of the aponeurosis of the internal oblique and the aponeurosis of the transversus abdominis muscle. Adapted from Gray's Basic Anatomy, 3rd edition, Drake RL, Vogl W, Mitchell AW, Abdomen, Pages 151–152, Copyright 2023, with permission from Elsevier [License Number 5664341396354] [53].

The TrA is the main component of most DRA protocols. These contractions are guided by an abdominal drawing-in maneuver (ADIM) following deep exhalation (Fig. 5). Researchers investigating TrA contractions via ultrasound, using the ADIM on DRA patients, found an immediate IRD increase, attributed to the muscle’s transverse fibers and pull angle [31, 36, 49–51] (Fig. 6), and tension of the LA minimizing distortion [12]. In a good-quality RCT [21], however, TrA exercises from various positions in DRA women showed a significant IRD decrease after 12 weeks, compared with only the taping or the control group (maintaining normal activity).

#### Co-Activation of Deep and Superficial Abdominal Muscles

In observational studies, researchers observed an IRD increase with TrA pre-activation during curl-up compared with no pre-activation [12, 49–51]. Lee and Hodges [12] observed an IRD increase during ADIM compared with curl-up and also noted LA distortion (anteriorly or posteriorly) during curl-up, which was reduced with deep and superficial abdominal co-contractions.

#### Eccentric Abdominal Muscle Training

Six studies [23, 33, 34, 42, 44, 47] proposed eccentric abdominal contractions in their rehabilitation protocols. Four of them [33, 34, 42, 44] included reverse sit-ups and trunk twists, along with other concentric exercises. Α good-quality RCT [23] included a modified eccentric-based sit-up from an upright seated position, which improved muscle strength and reduced abdominal bulging. Α fair-quality RCT [42] showed an IRD decrease in the group practicing eccentric exercises, whereas other good-quality RCTs [33, 34, 44, 47] applied eccentric exercises in both intervention and control groups.

#### Pelvic Floor Muscle Training

**Fig. 7:**
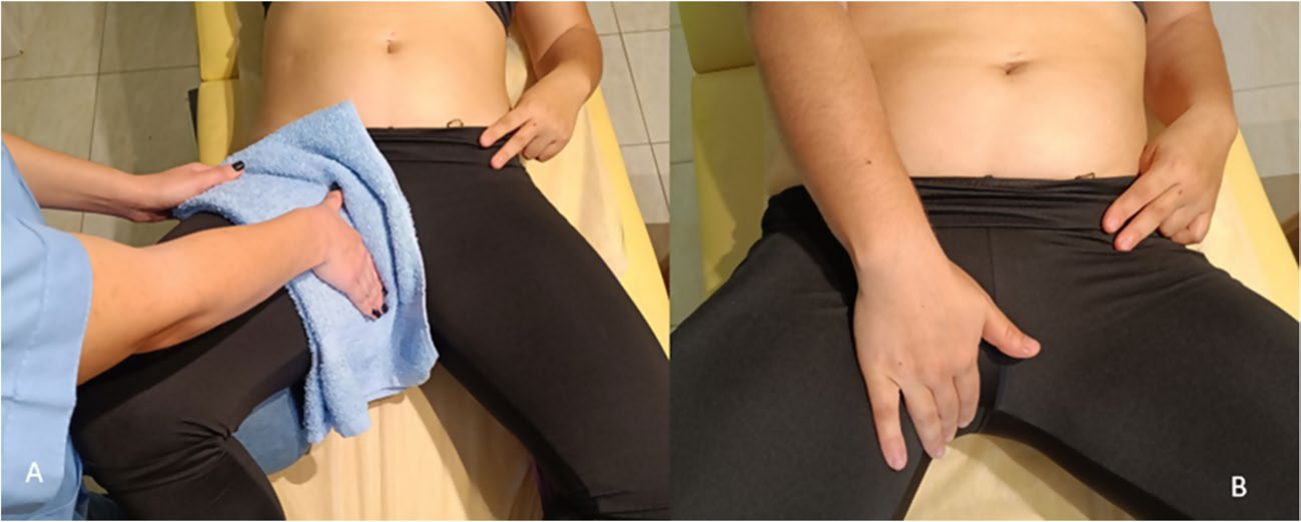
Activation of the pelvic floor muscles (PFMs). **A**. The patient is lying in a relaxed supine position with the legs bent, outwardly rotated and supported at the knees. They are then instructed to take a deep breath while relaxing the abdomen and the pelvic floor muscles and then to fully exhale while they contract the PFM. The physiotherapist uses instructions like “ tighten the muscles around the vagina and anus” or “tighten the muscles around the anus as if to stop passing wind and then gently draw the anus up towards the back of the pubic bone”. Palpation of PFM activation is performed using two fingers over the labia majora, laterally to the vaginal opening. A towel is commonly utilized for patient comfort during this procedure. **B**. The patient is guided to self-palpate the contraction of the PFM and maintain a contraction for 10 seconds (while breathing and audibly counting to 10)

Pelvic floor muscle training (Fig. 7) was applied in ten studies [22, 29, 32, 34, 39, 40, 42, 44, 45, 47]. Theodorsen et al. [31] found that both TrA and PFM increased IRDs, whereas TrA and PFM co-contracting resulted in the largest IRD increases. Gluppe et al. [36] found similar results infraumbilically. A good-quality RCT [34] applied electromyographic biofeedback-assisted pelvic floor muscle training (BAPFMT) along with abdominal exercises and neuromuscular electrical stimulation (NMES) of the RA and found decreased IRDs after 6 weeks compared with abdominal exercises and NMES only (control group).

#### Respiratory Maneuvers/Controlled Breathing

Eight studies [21, 32–34, 39, 44, 45, 52] utilized a respiratory maneuver at the beginning of abdominal exercises (such as drawing-in) or PFM exercises, to facilitate TrA and PFM contractions.

#### Functional Exercises

Eight studies included functional exercises such as planks [32, 39, 43–47], side planks [23, 37, 39], bridges [32, 37, 39, 45, 47], and squats [37, 39, 47]. IRD decreased significantly across groups performing such exercises. A good-quality RCT [44] found an exercise protocol containing planks to be superior to “traditional” abdominal exercises in decreasing IRD.

#### Alternative Exercise Therapy Interventions

Six studies proposed alternative exercises, such as electromyographic-biofeedback PFM exercises [34], suspension training [39], yoga [48], AHEs [22, 35], and low-impact aerobic and resistance training [32]. A fair-quality RCT [48] found that a yoga exercise program significantly reduced IRD postpartum, compared with a control group practicing no exercise. A good-quality RCT [39] proposed suspension training (suspension training system [STS]) with exercises from various positions against an isometric–isotonic core stabilization protocol proposed by Litos [54] and a non-active control group. STS reduced IRD but without significant differences between exercise groups. In an observational study [22] AHEs from a supine position narrowed IRD infraumbilically but tended to increase IRD supraumbilically. In a case series [35], a protocol containing AHEs, oblique, and RA exercises was successful in significantly reducing IRD after 9 weeks.

#### Other Interventions/Modalities

Nine studies used special modalities or equipment in their exercise protocols, proposing abdominal binding with garments [23, 42–44, 46] or bandages/scarves [33, 52] during exercise. In three RCTs [42, 43, 46] abdominal binding alone [42] or combined with stabilization exercises [43, 46] effectively reduced IRD in DRA women. Thabet and Alshehri [44] found that a core stabilization-focused exercise program combined with traditional abdominal exercises and abdominal binding was more effective than traditional abdominal exercises alone in reducing IRD and improving physical function. Similar effects were described in Keshwani et al.’s RCT [23], where trunk flexion strength was clinically meaningful at 12 weeks of intervention for the abdominal binding and combination therapy (exercise and abdominal binding) groups, for women starting the program at 3 weeks postpartum. In an observational study [41], abdominal binding reduced IRD during rest, but not during contractions (curl-ups and drawing-in maneuvers). In a good-quality RCT [21] adding elastic tape to the protocol did not improve exercise results, whereas abdominal binding was more effective in supporting the abdominal wall and decreasing IRD during rest in an observational study [41]. In two good-quality RCTs [33, 34], NMES was applied to promote maximal abdominal contractions and in combination with exercise reduced IRD and proved effective.

### Contribution of Rehabilitation Interventions to Other Outcome Measures

Apart from IRD reduction, DRA rehabilitation programs also decreased the waist/hip ratio and increased abdominal muscle efficiency (peak torque, maximum repetition total work, and average power) [23, 42], especially when they were combined with NMES [33] or abdominal binding [23]. In several RCTs, progressive and functional exercise programs have been shown to increase physical function [44], improve running speed for recreational runners with DRA [37], increase abdominal muscle thickness (RA, TrA, external and internal oblique), improve trunk endurance, reduce low back pain severity and disability [29], and health and functional areas of the maternal quality-of-life questionnaire [47]. PFM training using BAPFMT combined with abdominal exercises and NMES of the RA also improved physical functioning and quality of life (QoL) [34]. Progressive and functional exercise programs [32, 39] improved lumbopelvic control and proprioception impairment [39], low back pain intensity and disability [32, 39], static and dynamic balance [39], and stress urinary incontinence [32].

## Discussion

Overall, based on this scoping review, there is evidence that current rehabilitation practice is beneficial in several DRA-related and functional parameters for women postpartum. However, although researchers propose various exercises and adjunct modalities, there is no agreement on what constitutes the best approach.

### Evidence on Exercise Prescription

Observational studies support the notion that deep trunk muscle contractions are important in generating LA tension for supporting abdominal viscera and transferring forces across the midline of the abdomen. Researchers now agree that the ability to generate tension in the LA is crucial for abdominal wall function and is more important than complete DRA closure [12, 55, 56], whereas patient functionality, neuromuscular control, and muscle capacity to achieve force and form closure may be of greater clinical value [21]. Systematic reviews [24, 25] support encompassing TrA contractions in DRA rehabilitation programs, combined with upper and lower extremity exercises, trunk rotations, and functional training in various loading positions. Expert-based recommendations [56] report that optimal isolated and synergistic contractions of inner unit muscles and tension-free diaphragmatic breathing should be prioritized during the immediate postpartum period for DRA. Additionally, LA tension through TrA contraction is suggested to generate a connective tissue remodeling process, promoting DRA closure [23]. However, more research in this area is required.

Furthermore, a combination of deep and superficial abdominal contractions proved to be safe and effective in tensing the LA without further separating the rectus muscles [12]. Introducing trunk flexion exercises early postpartum is necessary, as most women perform RA-dominant tasks that increase abdominal pressure during daily activities and infant care. Retraining patients in generating and maintaining LA tension in static tasks with inner-unit and then combining them with outer-unit contractions in more dynamic exercises (such as trunk flexion and rotation) would be desirable. However, exercise progression should be individualized according to the patient’s needs, function, and progress. Interestingly, many protocols [29, 33–35, 42–44, 47, 52] introduce outer-unit targeting exercises (curl-ups, sit-ups, and trunk twists) early postpartum, without providing information about patients’ ability to generate tension in the LA. Moreover, in several protocols [21, 22, 29, 42, 44] no information is provided about exercise progression or adequate loading capacity. Future studies could include specific clinical/functional tests for assessing patients’ function and readiness to progress (maintain and transfer load) to more difficult exercises.

According to Dufour et al. [56] inner unit muscle retraining should also include PFM exercises, which are the gold standard approach to urinary incontinence [57] and are also proposed for DRA rehabilitation programs [58]. However, there is conflicting evidence regarding their effect on IRD [24, 34]. Observational studies [31, 36] have found PFM contractions almost as effective as TrA contractions for tensing the LA, whereas a co-contraction between them was found to be the most effective. Gluppe et al. [58], studying PFM training, found no significant differences in IRD reduction compared with control at 6 months post-intervention; however, the IRD reduction of the control group could have been due to spontaneous DRA resolution during early postpartum and not necessarily due to PFM ineffectiveness (as rehabilitation was initiated early). Nevertheless, adding electromyographic BAPFMT to abdominal exercises and NMES for RA was superior in decreasing IRD than abdominal exercises and NMES alone in an RCT [34], and it may be that muscle adaptations could be the result of sufficient PFM loading through electromyographic-biofeedback.

Various researchers have proposed eccentric abdominal contractions [23, 33, 34, 42, 44, 47]. Unfortunately, most protocols encompass eccentric exercises in both intervention and control [33, 34, 44, 47] groups; thus, no clear conclusions can be drawn. Eccentric muscle contractions are superior to concentric or isometric ones regarding muscular adaptations, such as strength [59], muscle mass [60], and structural arrangement [61] (muscle bundles’ length and pennation angles). Moreover, eccentric exercise can activate type II muscle fibers [59] and produce greater forces than concentric, resulting in easier load transfers [62]. Thus, eccentric contractions may be more suitable for the early postpartum period when abdominal muscles may be less effective at generating force. Although eccentric training has generally been extensively studied, its effect on the RA muscle or the DRA is as yet unknown. Indeed, researchers hypothesize that eccentric RA contractions might improve strength and muscle alignment, promote connective tissue remodeling, and reduce IRD [23, 47].

Several rehabilitation protocols encompass respiratory exercises, where diaphragmatic breathing or respiratory maneuvers are performed to facilitate a co-contraction of deep trunk muscles. Researchers suggest that the diaphragm contributes to spinal stability, working synergistically with TrA and PFMs to increase intra-abdominal pressure where necessary [63–65]. Several studies utilizing diaphragmatic exercises in patient populations and trunk dysfunctions reveal alterations in diaphragm morphology and function [66–70], correlations between diaphragm thickness and walking pace [71] or balance deficits [72, 73]; thus, supporting its trunk-stabilizing and functional role. Diaphragm parameters (thickness, excursion, etc.) have never been studied postpartum, and their role in DRA rehabilitation remains unknown, despite the strong recommendations for diaphragmatic rehabilitation for deeper abdominal muscle contractions [21, 32–34, 39, 44, 45, 52].

Alternative types of exercise, despite a scarcity of research, also appear effective. Suspension training was equally as effective as isometric–isotonic exercises for reducing IRD, improving lumbopelvic control and proprioception, static and dynamic balance, back pain, and disability [39]. Yoga was also effective in decreasing IRD in mild DRA [48]. AHEs were found to have a similar effect to a TrA contraction, increasing IRD and tensing the LA [22]. AHEs are believed to facilitate deeper abdominal and PFM contractions while reducing intra-abdominal pressure, relaxing the diaphragm, and elevating the pelvic viscera [22]. Two case series [35, 74] also reported promising results regarding IRD closure. However, it is suggested that AHEs should be prescribed with caution [75, 76] as TrA activation through AHEs may in fact increase intra-abdominal pressure, putting more strain on the PFM.

### Evidence on Adjunct Modalities

Several complementary interventions and modalities have also shown encouraging effects. Combined NMES with exercise significantly reduced IRD and increased abdominal muscle strength (peak torque, maximum repetition total work, and average power) compared with exercise alone [33].

There is some evidence that abdominal binding can reduce IRD and improve muscle function. These effects, however, cannot be attributed solely to abdominal binding, as in several studies patients also performed exercise [33, 43, 44, 46], and in others, patients were only a few days/weeks postpartum [23, 42] (where spontaneous resolution usually occurs). Some researchers speculate that abdominal support garments provide protection for collagen formation during exercise and support optimal muscle position for better performance and tissue remodeling [23]. Others, however, recommend their use with caution, as they may increase intra-abdominal pressure and put a strain on PFMs [57]. Currently, there is not enough evidence to support the use of elastic tape in DRA rehabilitation. Although taping may be helpful as a proprioceptive stimulus for deep abdominal contractions during activities that increase intra-abdominal pressure [21], abdominal binding is considered superior for abdominal wall support and IRD reduction compared with taping [41]. Considering the lack of evidence, we suggest that abdominal support garments are only introduced according to individual needs (i.e., following cesarean section, poor intra-abdominal control, etc.).

### Evidence on Other Outcomes

Exercise therapy was also effective in improving other outcomes and parameters. Low back pain severity and disability [29, 39], physical functioning, and QoL were improved in several studies [34, 44, 47]. Comparable results are presented in previous studies, where improvements in function, perceived pain [54], and quality of life were reported [19]. Exercise programs were also effective in improving stress urinary incontinence [32] and increasing running speed in recreational runners [37].

Exercise program efficacy seems to be independent of the rehabilitation setting. Supervised online exercise therapy was equally effective in increasing abdominal muscle thickness (RA, TrA, and obliques) and static trunk endurance as live sessions [47]. Moreover, unsupervised online therapeutic exercise (through videos) was also effective in reducing IRD, low back pain, and stress urinary incontinence [32]. However, unsupervised rehabilitation may prevent therapists’ ability to provide correcting cues and may also prevent compliance tracking.

### Limitations of the Studies Included

Methodological quality was relatively moderate across studies, with 8 of them graded as borderline good quality according to PEDro [21, 23, 33, 39, 44–47], making it challenging to draw definite conclusions. Sample sizes were also relatively low across studies, whereas only 2 undertook a prior power analysis [34, 46]. There was significant variability in inclusion criteria and assessment methods, with no consensus on predefined cut-off points for DRA diagnosis and assessment conditions (resting, head-lift, or curl-up). Researchers included women with variable DRA, ranging from minimal/borderline [21, 34, 37, 43, 45, 47, 48] to mild and severe [23, 35, 39] IRD, according to established classifications [1, 3], thus potentially biasing the effectiveness of rehabilitation. The timing of exercise initiation was variable, with women enrolling immediately after birth [40] to more than 6 months or 1 year postpartum [32, 35, 37, 43]. Early rehabilitation, however, could bias results owing to the natural resolution occurring during the first months postpartum [2, 4, 5], which could perhaps justify the IRD reduction across control groups [21, 40, 48], thus limiting the credibility of the studies. Nevertheless, greater IRD reductions favoring the intervention groups could indicate that focused exercise early postpartum could further enhance this natural effect. Exercise duration and frequency were also variable among studies. Although 2020 Swedish National Guidelines [17] recommend a 6-month rehabilitation training before considering surgery, guidelines on exercise duration for DRA are not well established. According to the American College of Sports Medicine Guidelines [77], strength training for healthy individuals should include at least 2–4 sets of 8–12 repetitions per set of 60–70% of one-repetition maximum (1-RM), for at least 2–3 days per week with progressive exercise intensity over time. Postpartum PFM training protocols also suggest at least 12 weeks’ duration for optimal muscle adaptations to occur [78]. However, several studies do not follow these guidelines [33, 34, 40], and only a third of them [23, 32, 37, 39, 43–48] propose progressions of isolated muscular and functional exercises (i.e., planks, bridges, squats, etc.), which are necessary for distinct neuromuscular, functional and fascial adaptations. Also, several studies [21, 22, 29, 35, 42, 44] fail to provide information about exercise prescription, such as training load and progression. Thus, it is reasonable to assume that the inability to reach statistical significance in DRA-related outcomes in some of these studies could be the result of a low training load and/or duration.

### Study Limitations

This scoping review was designed with the objective of systematically mapping rehabilitation interventions, applied to treat DRA during the postpartum period; however, it may have some limitations. In particular, the exclusion of non-English-language publications may introduce bias in study selection, whereas variability in diagnostic criteria, exercise program design, and assessment methods among the studies included may limit generalizability. Additionally, the lack of statistical comparison of results and the qualitative synthesis of findings may limit the reviewers’ ability to draw definitive conclusions. The inclusion of observational studies alongside clinical trials may further introduce biases and make it challenging to draw definitive conclusions about the long-term effectiveness of the interventions. Moreover, the review's focus on exercise interventions may overlook other potential approaches for DRA rehabilitation, such as manual therapy techniques or other adjunct therapies applied as a single treatment. The inclusion of observational studies [12, 30, 49, 51] that involve heterogeneous populations, such as pregnant and nulliparous women or men with DRA along with postpartum women, while complying with the predefined eligibility criteria (inclusion and exclusion), is a notable limitation of this scoping review. Although these studies may offer valuable insights for DRA patients from different populations, their inclusion could hinder the generalization of the results. The decision to include these studies was based on their potential contribution to the review's objectives, even though they did not exclusively focus on postpartum women with DRA. However, it is important to recognize this limitation while interpreting the outcomes of the review. Further research focusing specifically on postpartum women with DRA could help to provide more targeted and relevant evidence for clinical practice in this population. Despite these limitations, this scoping review serves as a valuable resource for understanding the current state of research on DRA rehabilitation postpartum and identifying areas for further investigation and evidence-based practice.

### Implications for Research

The identified limitations in the included studies lead to important implications for future research in postpartum DRA rehabilitation. Future studies should focus on rigorous designs, adequate sample sizes, and standardized diagnostic tools and criteria to strengthen the evidence. Prioritizing functional outcomes as well as IRD reduction and adhering to muscle-strengthening principles in exercise protocols is essential. Most importantly, aligning with expert recommendations and guidelines will standardize research practices and lead to more robust evidence for clinical decision making.

Additionally, research including women over 6 months postpartum may be essential for advancing our understanding of effective DRA rehabilitation. Interestingly, access to postpartum rehabilitation services varies widely around the world. In some health care systems, women can start rehabilitation after medical clearance, often around 6 weeks postpartum, whereas in other systems, rehabilitation is not standard unless a woman reports a specific issue, leading to delayed access. However, women tend to seek help for persistent dysfunction, especially upon returning to pre-pregnancy activities, which can extend up to 6 or more months postpartum. Understanding DRA recovery beyond 6 months post-birth, a timeframe more realistic in various regions, is crucial. This perspective provides a clearer view of the true effects of exercise and adjunct interventions by eliminating the concurrent impact of spontaneous resolution, thus aiding researchers and clinicians in improving their ability to provide accurate recommendations and guidelines. Specific guidelines and clinical recommendations are vital, particularly in regions with limited access to post-birth rehabilitation. Enhancing communication and collaboration among researchers and health care professionals can expedite women's access to essential women’s health services.

### Clinical Implications

This review suggests that exercise therapy, including abdominal strengthening and deep trunk stabilizer exercises, can effectively reduce IRD and improve DRA-related dysfunction postpartum. However, interventions should also be aimed at improving muscle strength and functional ability because full IRD recovery may not always occur. Proper intra-abdominal pressure management is critical, and patients should be taught to engage their deep trunk muscles during exercise and daily activities. Health care professionals should adhere to evidence-based training principles and consider incorporating various modalities such as electromyographic biofeedback, NMES, abdominal binders, or taping to optimize outcomes. Timing of rehabilitation interventions should also be considered, and individualization of rehabilitation programs based on DRA severity, individual goals, and possible coexisting dysfunctions is crucial. Further research is needed to better understand the mechanisms by which exercise may reduce IRD and delineate specific exercise prescription parameters for DRA rehabilitation.

Additionally, in this review we seek to provide insights to clinicians specializing in women's health regarding available options for the rehabilitation of DRA. Women with DRA are usually underdiagnosed, resulting in many patients being recommended by nonqualified professionals to engage in nonspecific physical exercises that may further strain the abdominal wall. Furthermore, there is a misconception that DRA is primarily a cosmetic issue, underestimating its functional impact or potential associated dysfunctions [79]. A recent study revealed that women who were concerned with their abdominal appearance post-partum are most likely to seek advice on treatment through social media [80]. Improved communication among health care professionals is crucial for providing women with access to evidence-based women's health services. Physicians involved in women’s health, obstetricians, and urogynecologists should be well-informed about evidence-based rehabilitation interventions, suggesting conservative and effective solutions, guiding women to make informed choices and avoid unnecessary invasive procedures when possible.

## Conclusions

This scoping review provides valuable insights into postpartum DRA rehabilitation, suggesting that exercise therapy might be an effective approach for treating DRA and improving overall function. However, the study also sheds light on several limitations, including methodological variability, heterogeneous inclusion criteria, and lack of adherence to evidence-based exercise principles. These limitations highlight the need for more standardized and rigorous research on this topic. Additionally, further investigation is required to understand the mechanisms of IRD reduction and establish specific exercise prescription parameters. Health care professionals should consider a more holistic but still individually tailored approach to DRA reduction, including progressive functional exercises, breathing exercises with PFM training, and individualized inner- and outer-unit abdominal training. By addressing these gaps, future research can contribute to evidence-based guidelines that enhance the overall quality of care for postpartum women with DRA.

### Supplementary information

ESM 1 (DOCX 10563 kb)

## Appendix 1: search strategy and results

### Search strategy PubMed (MEDLINE)

1. Topic diastasis recti abdominis: "Diastasis, Muscle"[Mesh] OR ("diastasis recti"[Text Word]) OR ("rectus abdominis diastasis"[Text Word]) OR ("abdominal separation "[Text Word]) OR (DRA[Text Word]) OR (DRAM[Text Word]) OR ("diastasis recti") OR ("rectus abdominis diastasis") OR ("abdominal separation ") OR (DRA) OR (DRAM) OR ("inter-recti distance")

2. Topic physiotherapy: "Exercise Therapy"[Mesh:NoExp] OR (physiotherapy[Text Word]) OR ("physical therapy"[Text Word]) OR (exercise*[Text Word]) OR (rehabilitation[Text Word]) OR ("conservative treatment"[Text Word]) OR (physiotherapy) OR ("physical therapy") OR (exercise*) OR (rehabilitation) OR ("conservative treatment") OR (physiotherap*) OR (program*) OR (training) OR (contraction*) OR ("conservative treatment")

3. Topic postpartum: "Postpartum Period"[Mesh:NoExp] OR (postpartum[Text Word]) OR (parous[Text Word]) OR (postpartum[Text Word]) OR (parous[Text Word]) OR (postpartum) OR (parous)
4. #1 AND #2 AND #3

## Appendix 2

Table 3

**Table 3:** Characteristics of the studies included

Study	Study design/aim of the study	Sample size/age/sample profile	Delivery mode/parity mode/time postpartum	Intervention group/control group	Frequency/progression/duration of intervention	Assessment time points/follow-up	Adherence/drop-outs	Outcome measures/IRD assessment tool	Results
RCTs									
El-Mekawy et al. [42]	RCT/effectiveness of intervention on IRD, waist circumference, waist/hip ratio, abdominal muscle strength	N=30 (group A: 15, group B: 15)/age: 25–35/postpartum women, DRA cut-off points not reported (group A baseline IRD: 3.01±0.14 cm; group B baseline IRD: 3.10±0.26 cm)	Vaginal delivery/primiparous/ 2nd day pp	Group A: abdominal belt. Group B: abdominal exercise program (static abdominal contraction, posterior pelvic tilt, reverse sit-up exercise, trunk twist, and reverse trunk twist)	5-s contraction, 10-s relaxation, 20 reps per exercise/3× per week and daily abdominal exercises at home/no progression reported/6 weeks total	At baseline and after 6 weeks/no follow-up	Drop-outs not reported/adherence not reported	Waist circumference, waist/hip ratio, IRD, abdominal muscle strength (peak torque, maximum repetition total work, and average power through isokinetic dynamometry)/IRD assessed by dial caliper just above the umbilicus during a curl-up	Statistically significant (*p*<0.05) decrease in all outcome measures for both groups. Statistically significant (*p*<0.001) decrease in IRD in favor of group B. Statistically significant waist/hip ratio decrease (*p*<0.01) in favor of group B. Statistically significant increase (*p*<0.001) in abdominal muscle efficiency (peak torque, maximum repetition total work and average power) in favor of group B
Walton et al. [43]	RCT/effectiveness of intervention on IRD, disability, pelvic floor dysfunction	*N* = 9 (intervention group, *n*= 5; “rraditional group”, *n*= 4)/age: 18–45/postpartum women with DRA—cut-off points not reported (intervention group IRD=8.75±0.87 mm; “traditional group” IRD=10.97±1.96 mm)	Cesarean section (*n*=1) and vaginal delivery (*n*=8)/parity not reported/3 months to 3 years pp	Intervention group: plank (10 s on knees or toes)/«Traditional» training group: abdominal crunch, both programs contained; posterior pelvic tilt, PFM exercises (kegels), exercises for oblique abdominals (Russian twist), use of abdominal binding during exercise	3x10 reps 10s plank, 3x per week and HEP 3x per week/repetitions increased to personal fatigue level/6 weeks total	Baseline and at 6 weeks/no follow-up	Total drop-out: 1, adherence: not reported	IRD, ODI, PFDI/ultrasound and caliper 4.5 cm above, at, and 4.5 cm below the umbilicus during a curl-up	Post-test: experimental: IRD: 7.58±2.02 mm. Traditional:IRD: 6.63±1.65 mm. Statistically significant difference at the umbilicus for both groups, with the traditional group showing greater closure after treatment. No statistically significant differences between groups (*p*=0.208) and below the umbilicus (*p*=0.674). ODI: no significant difference between groups (*p*=0.569). PFDI: no significant difference between groups (*p*=0.510). Significant difference in the CRADI scores within both groups (pre-test to post-test; *p*=0.042)
Kamel & Yousif [33]	RCT/effectiveness of intervention on IRD, abdominal muscle strength	*N*=60 (intervention group, *n*=30; control group, *n*= 30)/age: group A, 29.33±2.98; group B, 29.50±3.00/postpartum women with DRA >2.5 cm (group A baseline IRD: 2.86±0.31 cm; group B baseline IRD: 2.82±0.28 cm)	Vaginal delivery/primi- and multiparous/2 months pp	Group A (intervention group): abdominal exercise + NMES of rectus abdominis (NMES was applied first, followed by the abdominal exercises)/group B (control group): abdominal exercise with abdominal binding (scarf). Abdominal exercises: (sit-up, reverse sit-up, reverse trunk twist, u-seat, respiratory maneuver + TrA activation)	20 reps, 3× per week, and HEP daily/(abdominal exercises progressed by 4 reps per week), respiratory maneuver of 5 reps ×1 set (progressed by 1 set/week)/8 weeks total	Baseline and at 8 weeks/no follow-up	Total drop-out: 3 (group A, *n* = 1; group B, *n*=2)/analysis of patients who finished all sessions	IRD, abdominal muscle strength (isokinetic)/IRD assessed by ultrasound at X-U/2 and U-P/2 during rest	Post-test: group A: IRD: 1.43±0.38cm; group B: IRD: 2.09±0.35cm. Significant difference in decrease of IRD between groups in favor of group A: −0.65 (95% CI: −0.85, −0.46). Abdominal muscle strength: significant difference in group A vs group B in peak torque (N/m): 5.22 (95% CI: 1.95, 8.5
Tuttle et al. [21]	RCT/effectiveness of intervention on IRD, pelvic floor dysfunction, disability	*N*=30 (TRA training *n*=10; tape *n*=8; TRA + tape *n*=5; minimal intervention group *n*= 7)/age: 32.03±4.3/postpartum women with DRA (palpable separation ≥2 finger widths), baseline IRD. At rest: TRA exercise + taping group IRD: 2.70±0.19 cm; TRA exercise–alone group IRD: 2.30±0.49 cm; taping-alone group IRD: 2.36±0.68 cm; control group IRD: 2.29±0.66 cm	Delivery mode not reported/primi-and multiparous/6−12 weeks pp	TRA training (*n*=10), tape (*n*=8), TRA + tape (*n*=5)/minimal intervention group (*n*=7) instructed to maintain a normal level of activity	10 reps, 4−5× per week/progression not reported/12 weeks’ duration	At baseline and at 12 weeks/no follow-up	Total drop-out: 3 TRA (*n* = 1), TRA + tape:(*n* = 1), tape (*n* = 1)/adherence: average all groups: 79%, TRA training only: 95%	IRD, PFDI-20, RDQ/assessed by ultrasound 4.5 cm above and below the umbilicus during rest and head-lift	IRD during rest: TRA exercise + taping group: 1.39±0.29 cm; TrA training: IRD:1.34±0.37 cm; taping-alone group: 1.92±0.44 cm; minimal intervention group: 2.1±0.99 cm. Close to a significant difference in IRD between groups: −0.76 (95% CI: -1.53, 0.01). Significantly greater decrease in IRD at rest and during head-lift in the groups with TRA training compared with control/tape. PFDI-20: no significant difference between groups (*p* >0.05). RMDQ: no significant difference between groups (*p* >0.05)
Thabet & Alshehri [44]	RCT/effectiveness of intervention on IRD and function	*N*=40 (group A, *n*= 20; group B, *n*0)/age: 22−35/postpartum women with DRA (cut-off points not reported), group A baseline IRD: 28.35±1.04 mm, group B baseline IRD: 28.50±0.95 mm	Parity and delivery mode not reported/3−6 months pp	Group A: deep core stability-strengthening program (use of abdominal binding, diaphragmatic breathing, PFM exercises, planks, and isometric abdominal contraction) + traditional abdominal exercise program/group B: traditional abdominal exercises (static abdominal contractions, posterior pelvic tilt, reverse sit-up, trunk twist and reverse trunk twist	Both groups: 3× 20 reps, 5-s contraction hold, 10-s relaxation, 3× per week and daily at home/progression not reported/8 weeks in total	At baseline and at 8 weeks/no follow-up	No drop-outs/adherence: not reported	IRD, PF-10/IRD assessed by caliper 4.5 cm above the umbilicus during a modified sit-up	Post-intervention: Deep core program: IRD: 2.01±0.07 cm; traditional exercises: IRD: 2.37±0.11 cm. Significant difference in IRD between groups in favor of group A (MD: −0.36 (95%CI: −0.42, −0.30). Significant difference in PF-10 for group A compared with group B (MD: 5.25, *p*=0.0001)
Keshwani et al. [23]	Pilot RCT/effectiveness of intervention on IRD, abdominal muscle strength, pelvic floor dysfunction, body image, function	*N*=32 (exercise therapy *n*.=8; abdominal binding *n*8; combination therapy *n*=8; minimal intervention group *n*=8)/age: 31±3/postpartum women with DRA (palpable separation ≥2 finger widths), exercise therapy group: 2.54±0.59 cm, abdominal binding group: 3.80±1.38 cm, combination group: 2.57±1.92 cm, minimal intervention group: 3.00±1.38 cm	Vaginal delivery/primiparous/3 weeks pp	Exercise therapy group (*n*=8): weekly individualized sessions and daily home exercise including isolated activation of TRA/abdominal binding group (*n*=8): binding during waking hours/combination therapy group (*n*=8): combination of exercise therapy and abdominal binding/minimal intervention group (*n*=8): no intervention or education. Home program: isolated activation of the transversus abdominis muscles, bent knee leg lifts in crook lying while maintaining a neutral lumbopelvic spine, eccentric trunk flexion exercises starting in a sitting position using a sheet/towel to support the abdominal wall during the exercise, and side planks with progressive variations	3× 10 repetitions, 7× per week/progressive variations of exercises/12 weeks in total	At baseline and at 12 weeks/follow-up at 6 months	6 months total drop-out: 5; exercise therapy (*n*=2), control (*n*=1), exercise therapy + abdominal binding (*n*=2)/adherence: exercise therapy, 73% (home exercise) and 10/12 of the weekly sessions; abdominal binding, 60%; combination group, similar to the interventions delivered alone	IRD, abdominal muscle strength, PFDI, MBSRQ, IFSAC/assessed by ultrasound at the umbilicus, at 3 cm above, at 5 cm above, and 3 cm below the umbilicus during rest	Post-intervention: 6 months exercise therapy: IRD: −0.93±0.88 cm. Abdominal binding: IRD: −1.34±0.34 cm. Combination: IRD: −1.24±0.73 cm. Minimal intervention: IRD: −1.31±1.08 cm; no significant difference between groups. Exercise therapy vs minimal intervention, no significant difference between groups (MD: −0.38, 95%CI: −1.45, 0.68). Abdominal muscle strength: positive effects (Cohen`s d(d)=0.5−0.7) in the exercise and combination groups. PFDI: no effects in any groups. Body image: positive effects (d=0.2−0.5) in the abdominal binding alone and combination groups. IFSAC: no effects (d=0.0−0.3) in any groups
Laframboise et al. [45]	RCT/effectiveness of intervention on IRD, weight, muscle endurance	*N*=8 (intervention and control sample size not reported)/age: 35.6±3.2/postpartum women with DRA (cut-off points not reported), intervention group baseline IRD 33.2±3.8 mm; control group baseline IRD 28.5±10.0 mm at the umbilicus during rest	Delivery mode not reported/primi-and multiparous, singleton/6−24 months pp	Intervention group: online course containing core exercises, breathing techniques, PFM contractions and mindfulness teachings, glute bridges, planks, etc./control group: no intervention	3× per week/program complexity progressed/12 weeks intotal	At baseline and at 12 weeks/no follow-up	Total dropout: 1/adherence not reported	IRD, weight, UHBE/IRD assessed by digital caliper at the umbilicus, 4.5 cm above and 4.5 cm below the umbilicus in two phases (rest and active)	IRD: significant group × time interaction: 2 inches above the umbilicus (rest) (*p*=0.007, d=0.67) and 2 inches above the umbilicus (active; *p*=0.005, d=0.69); nonsignificant group × time interaction for weight (*p*=0.06, d=0.23). Nonsignificant group × time interaction for core function (*p*=0.83)
Pompolim et al. [40]	RCT/effectiveness of intervention on IRD	*N*=50 (treatment group, *p*=25; control group, *n*2)/age: 22.6±3.28/postpartum women with DRA >3 cm during a curl-up (treatment group baseline IRD at 3 cm above the umbilicus: 36.0±2.7 mm; control group baseline IRD at 3 cm above the umbilicus: 34.5±2.8 mm)	Vaginal delivery/primi- and multi-parous/6 h after delivery	Treatment group (intervention group): hip adduction with pillow + PFM contraction, TrA contraction, twisted curl-up/control group: no intervention	2 sessions (at 6 h and at 18 h)/10 reps during the first session, and 20 reps during the second session	At 6 h and at 18 h after delivery	Drop-outs and adherence not reported	IRD/assessed with a caliper at 3 cm above and at 3 cm below the umbilicus during a curl-up	IRD reduction for the control group: supra-umbilical (MD: −0.6±0.4, *p*<0.001), infra-umbilical (MD: -0.8±0.7, *p*= 0.015); IRD reduction for treatment group: supra-umbilical (MD: −1.4±0.4, *p*<0.001), infra-umbilical (MD: −0.8±0.3, *p*<0.001); IRD reduction for the treatment group was significantly greater above the umbilicus (*p*<0.001) compared with the control group. No significant difference in IRD reduction below the umbilicus between the treatment group and the control group (*p*=0.55)
Saleem et al. [29]	RCT/effectiveness of intervention on IRD and disability	*N*=40 (group A, *n*=20; group B, *n*=20)/age: group A: 29.8±4.1; group B: 30.2±4.3/postpartum women with DRA (≥2 cm), group A baseline IRD: 27.89±1.69 mm; group B baseline IRD: 27.18±1.28 mm (during a curl-up, assessment point not reported)	Vaginal delivery or cesarean section/parity not reported/3 months pp	Group A: abdominal crunches, static glutei, kegels, and isometric back strengthening exercises/group B: double straight leg raise, static glutei, kegels, and isometric back-strengthening exercise	3×10 reps per session, 3× per week/progression not reported/6 weeks in total	At baseline and at 6 weeks/no follow-up	Drop-out and adherence not reported	IRD, ODI/IRD assessed by (a)finger palpation method, at the umbilicus, 2 cm above the umbilicus and 2 cm below the umbilicus, (b)digital nylon caliper at the umbilicus, 4.5 cm above the umbilicus and 4.5 cm below the umbilicus during a curl-up	IRD mean difference via finger palpation method: group A: (1.95±0.18, *p*<0.001), group B: (2.85±0.35, *p*=0.046). IRD mean difference via digital nylon caliper method: group A: (4.06±0.74, *p*<0.001), group B: (1.2±0.49, *p*<0.001). Statistically significant differences between groups (*p*<0.001). ODI mean difference: group A: (2.70±1.05, *p*<0.001), group B: (1.1±0.06, *p*<0.001). Statistically significant differences between groups (*p*<0.001)
Awad et al. [46]	RCT/effectiveness of intervention on IRD	*N*=50 (group A, *n*=25; group B, *n*=25)/age: 23−35/postpartum women with DRA (IRD cut-off point not reported), baseline IRD of both groups: 29.69±1.61 mm at 4.5 cm above the umbilicus during rest	Vaginal delivery/parity level not reported/3−6 months pp	Group A (intervention group): progressive prone plank exercise program + belly band + advice/group B (control group): belly band + advice	1-min hold, 3× per week and HEP regularly/progressively up to 20 reps × 3 sets, moderately hard perceived exertion/8 weeks in total	Baseline and at 8 weeks/no follow-up	No drop-outs/adherence not reported	IRD/ultrasound assessment at rest and during contraction of the RA at 4.5 cm above and below the umbilicus	Post-treatment: statistically significant reduction of IRD for both groups at 4.5 cm above and 4.5 cm below the umbilicus, during rest and RA contraction. IRD at rest and during RA contraction reduced significantly more for group A compared with group B at 4.5 cm above the umbilicus (*p*=0.001) and 4.5 cm below the umbilicus (*p*=0.001)
Kim et al. [47]	RCT/effectiveness of intervention on IRD, abdominal muscle thickness, trunk endurance, quality of life	*N*=37 (online group: *n*=19; offline group *n*1)/age: online group, 31.68±3.92; offline group, 32.72±2.54/postpartum women with DRA (cut-off point not reported), online group baseline IRD: 1.99±0.26 cm; offline group baseline IRD: 1.92±0.30 cm	Vaginal delivery or cesarean section/parity not reported, singleton/6 months to 1 year pp	Online group: 40-min trunk stabilization exercise protocol/offline group: same program in person	40-min sessions, 2× per week and recommended to do the exercises as often as possible/progression of reps and sets as reported on Litos [54]/6 weeks in total	At baseline and at 6 weeks/no follow-up	Drop-outs: online group *n*=1, offline group *n*=2/adherence not reported	IRD, abdominal muscle thickness (ultrasound), static trunk endurance (Torso endurance test (trunk flexor endurance test and bilateral side bridge tests), MAPP-QOL/IRD measured by ultrasound at 2.5 cm above the umbilicus at rest	IRD (cm): online group post-test: 1.37±0.40, MD post-test: −0.62±0.27 (*p*<0.001). Offline group post-test: 1.18±0.30, MD post-test: −0.74±0.23 (*p*<0.001). Between groups MD post-test: 1.471 (*p*>0.05). Abdominal muscle thickness: significant increase in EO, IO, TrA, and RA post-test in both groups (*p*<0.001). MD between groups only significant for left RA in favor of the offline group (−2.599, *p*=0.014). Static trunk endurance: significant increase in all tests post-test in both groups (*p*<0.001), MD between groups only significant for right side bridge in favor of the offline group (−2.278, *p*=0.029). MAPP-QOL: the greatest improvement was observed in health and functional areas for both groups (*p*<0.001), no significant difference between the two groups (*p*>0.05), significant improvement in the online group (*p*<0.001) in the spouse category compared with the offline group (*p*<0.05)
Liang et al. [34]	RCT/effectiveness of intervention on IRD and function	*N*=66 (study group: *n*=33, control group: *n*=33/age 29.9±4.3/postpartum women with DRA (IRD >2.5 cm, 2 cm above or 2 cm below the umbilicus), study group baseline IRD: 2.8±0.9 cm; control group baseline IRD: 2.9±0.7 cm	Vaginal delivery/primi- and multiparous, singleton/2−6 months pp	Study group: abdominal exercises (Kamel & Yousif [33] protocol) + electromyographic BAPFMT + NMES of the rectus abdominis/control group: abdominal exercises (Kamel & Yousif [33]) protocol) + NMES of the rectus abdominis	Study group: abdominal exercises 1× per week (supervised); HEP daily; BAPFMT (20’ per session)+ NMES of the rectus abdominis muscles (20’ per session) 3× per week/control group: abdominal exercises 1× per week (supervised) and HEP daily, NMES of the rectus abdominis muscles (20’ per session) 3× per week/progression of abdominal exercises by reps and sets as reported in Kamel & Yousif [33]/6 weeks in total for both groups	At baseline and at 6 weeks/no follow-up	Drop-out: 1 (control group)/adherence 30/33 in the study group, 30/33 in the control group	IRD, SF-36-PCS and MCS/IRD measured by ultrasound at 2 cm above or 2 cm below the umbilicus	IRD: study group at 6 weeks: 1.6 (±0.3)cm, control group at 6 weeks: 2.0 (±0.3) cm, MD at 6 weeks: −0.4 (95% CI: −0.59 to −0.26, *p*<0.001) in favor of the study group. SF-36 PCS: study group at baseline: 40.1 (±3.8), study group at 6 weeks: 45.5 (±1.2), control group at baseline: 39.7 (±5.4), control group at 6 weeks: 41.2 (±2.6), MD at 6 weeks: 4.3 (95% CI: 3.72–4.50, *p*<0.001) in favor of the study group. SF-36 MCS: study group at baseline: 64.9 (±7.2), study group at 6 weeks: 68.6 (±11.3), control group at baseline: 68.3 (±11.4), control group at 6 weeks: 72.4 (±6.7), MD at 6 weeks: −3.8 (95% CI: −4.17 to −3.25, *p*>0.05)
Yalfani et al. [39]	RCT/effectiveness of intervention on IRD, lumbopelvic proprioception and control, low back pain and disability	*N*=36 (STS group, *n*=12; ISoM−ISoT group, *n*=12; control group, *n*=12/age: 29.11±4.85/postpartum women with DRA (IRD >20 mm at 4.5 cm above the umbilicus during a curl-up), STS group baseline IRD: 42.5±10.77 mm, ISoM−ISoT group baseline IRD: 36.33±7.19mm, control group baseline IRD: 38.01±9.14 mm	Vaginal delivery/multi-parous/65.78±28.29 days pp	STS group: suspension exercises from various positions, ISoM−ISoT (core stabilization exercises) group: core stabilization exercises according to Litos [54] protocol/control group: no exercise	50-min sessions, 3× per week/progression of exercises every 6 sessions (2 weeks)/8 weeks in total	At baseline and at 8 weeks/no follow-up	Dropouts: *n*=9/good adherence if 22/24 sessions complete	IRD, lumbopelvic proprioception impairment (goniometer), lumbopelvic control impairment (lateral step-down test), postural static and dynamic instability (Biodex balance system), low back pain (VAS scale), disability (ODI)/IRD measured by digital caliper at 4.5 cm above the umbilicus during a curl-up	IRD: STS vs ISoM−ISoT MD: −2.76 (*p*=0.12), STS vs control MD: −21.65 (*p*=0.001, η^2^=0.874). ISoM−ISoT vs control MD: − 18.89 (*p*=0.001, η^2^=0.846). Lumbopelvic proprioception impairment: statistically significant differences in STS vs control (MD: −7.83, *p*=0.001, η^2^= 0.818) and ISoM−ISoT vs control (MD: −7.33,*p*= 0.001, η^2^=0.850). Lumbopelvic control impairment: statistically significant differences in STS vs control (MD: −10.68, *p*= 0.001, η^2^=0.812) and ISoM−ISoT vs control (MD: −12.21, *p*=0.001, η^2^=0.851). Postural static instability (Biodex balance system): statistically significant differences in STS vs control (MD: −0.39, *p*=0.013, η^2^=0.190) and ISoM−ISoT vs control (MD: −0.47, *p*=0.005, η^2^=0.252). Postural dynamic instability: statistically significant differences in STS vs control (MD −0.63, *p*=0.032, η^2^= 0.152) and ISoM−ISoT vs control (MD: −0.96, *n*=0.004, η^2^=0.262). Low back pain (VAS scale): statistically significant differences in STS vs control (MD: −2.83,*p*=0.001, η^2^= 0.669) and ISoM−ISoT vs control (MD: −2.31,*p*=0.001, η^2^= 0.546). Disability (ODI): statistically significant differences in STS vs control (MD: − 17.57,*p*=0.001, η^2^= 0.671) and ISoM−ISoT vs control (MD: −15.83, *p*=0.001, η^2^=0.720)
Li et al. [48]	RCT/effectiveness of intervention on IRD	*N*=129 (yoga exercise group, *n*=65; control group, *n*=64)/age 30−31/postpartum women (IRD at 3 cm above the umbilicus at 6 weeks: control group: 2.45±0.82 cm; yoga exercise group: 1.93±0.76 cm), DRA cut-off point not reported)	Vaginal delivery/primiparous/1−12 weeks pp	Yoga exercise group: core breathing and yoga postures (cat style, tiger style, half ship style, simple side plate style, antiquadruped style, etc.)/control group: no intervention	60 min sessions, 1× per week/exercise progression according to week: divided into two periods: (1) postpartum weeks 1−6 (1−1, 1−2, 1−3, and 1−4); (2) postpartum weeks 6−12 (2−1, 2−2, 2−3, and 2−4)/12 weeks in total	At 6 weeks and at 12 weeks pp/no follow-up	Drop-outs: yoga exercise group, *n*=2; control group, *n*=11/adherence not reported	IRD/IRD measured by ultrasound at the umbilicus and 3 cm above and below the umbilicus during rest	Weeks 6−12: yoga exercise group: statistically significant reduction of IRD at 3 cm above the umbilicus (MD: 0.56±0.45, *p*<0.001), at the umbilicus (MD: 0.65±0.50, *p*<0.001), and 3 cm below the umbilicus (MD: 0.39±0.32, *p*<0.001). Control group: statistically significant reduction in IRD at 3 cm above the umbilicus (MD: 0.19±0.60, *p*= 0.024) and at 3 cm below the umbilicus (MD: 0.15±0.48, *p*= 0.006). Weeks 1−6: the yoga exercise group IRD reduced significantly more at 3 cm above the umbilicus (*p*<0.001) and at the umbilicus (*p*<0.01) compared with the control group IRD. Weeks 6−12: yoga exercise group IRD reduced significantly more at 3 cm above the umbilicus (*p*<0.001) and at the umbilicus (*p*<0.001) compared with the control group IRD
Case studies and case series									
Hanif [52]	Case series/effectiveness of intervention on IRD	*N*=3/age: 27−33/severe DRA (IRD >7 cm cut-off point not reported)	Vaginal delivery, *n*=2; Cesarean section, *n*=1/primiparous, *n*=2; multiparous, *n*=1/>6 months pp	Therapeutic exercise, *n*=2; modified Noble’s reduction exercise (head-lift with abdominal support)/no intervention, *n*=1	3- to 5-s hold, 50 reps/day, 3× per week/progression not reported/6 weeks total	Baseline and at 6 weeks/follow-up assessment after 16 weeks	No drop-out/adherence not reported	IRD/measured by caliper at the umbilicus during a head-lift	IRD baseline: exercise group: case 1: IRD=8 cm , case 2: IRD=10.4 cm; no exercise: case 3: IRD=7 cm. IRD at 6 weeks: exercise group: case 1: IRD=2.0 cm, case 2: IRD=4.8 cm; no exercise: case 3: IRD=7 cm. IRD at follow-up: exercise group: no change, no exercise: case 3: IRD=7.8 cm
León et al. [35]	Case series/effectiveness of intervention on IRD	*N*=100/age 39.25±4.76/postpartum women with DRA (IRD ≥2.5 cm), medial IRD at baseline: 4.02±1.21 cm	Vaginal delivery and cesarean section/primi- and multiparous/2 months to 35 years pp	Hypopressive exercises, oblique and RA activation/no comparison	1× per week and daily exercises at home through an online platform/progression according to patient performance/9 weeks total	At baseline, at 3, 6, and 9 weeks/no follow-up	Drop-outs and adherence not reported	IRD/measured by caliper at the umbilicus, 4 cm above, and 4 cm below the umbilicus during a head-lift	Weeks 0−3: significant reduction of IRD at all three sites (*p*=0.00). Weeks 3−6: significant reduction of IRD at all three sites (*p*=0.00). Weeks 6−9: significant reduction of IRD at all three sites (*p*=0.00)
Deering et al. [37]	Case series/effectiveness of intervention on IRD, muscle activation, average running distance and speed, running gait variables	*N*=13/age: 32.8±2.7/parous recreational runners (DRA cut-off point not reported), baseline IRD 2.3±0.7 cm	Cesarean section and vaginal delivery/primiparous and multiparous/7 weeks to 2 years pp	Abdominal muscle retraining program and HEP (abdominal drawing-in maneuver and stabilization exercise, side plank, squats (double-leg, unilateral)/no comparison	Abdominal drawing-in and stabilization exercise: 10 s, 10 reps, 1× per day, side plank: 10 s, 6 reps each side, squats: (double leg: 10 reps, 2 sets, 1× per day, unilateral: 20 reps, 2 sets each leg, 3× per week), one supervised session per week, and HEP daily/weekly progression of exercises/8 weeks in total	At baseline and at 8 weeks (end of intervention)/follow-up 6 weeks after intervention	Drop-outs: 3/adherence not reported	IRD, IO, and TrA activation ratio, average running mileage per week, average running speed, primary running gait variables (pelvic drop excursion, pelvic rotation excursion, peak hip adduction during stance)/IRD measured by ultrasound at 2.5 cm above and below the umbilicus during rest	IRD decreased significantly below the umbilicus: baseline: 1.8±0.9, end of intervention: 1.2±0.6. IRD at 6-week follow-up: 1.1±0.6 (*p*=0.006, η^2^=0.602), average running speed increased from study baseline to end of intervention (*p*=0.021), no change in running mechanics, IO and TrA activation ratio, and average running mileage per week
Observational studies									
Pascoal et al. [49]	Preliminary case−control study/ultrasound assessment during tasks	*N*=20 women (postpartum women, *n*=10; nulliparous women, *n*=10)/age: 30±4/DRA cut-off points not reported, postpartum women resting IRD: 14.67±3.14 mm, nulliparous women 9.63±2.84	Vaginal delivery, *n*=9; cesarean section, *n*=1/primiparous/1−5 months pp	Measurements at rest and during abdominal crunch	Single test	Single test	Not reported	IRD/ultrasound assessment at 2 cm above the umbilicus	Curl-up vs rest: postpartum women IRD: 10.7±3.1 mm. Nulliparous women IRD: 13.38±3.11 mm
Sancho et al. [50]	Cross-sectional study/ultrasound assessment during tasks	*N*=38/age: vaginal delivery group 31.2±3.6, cesarean section group: 32.3±4.4/postpartum women (DRA cut-off points not reported), vaginal delivery group IRD: 25.5±9.0 mm, cesarean section group IRD: 26.5±9.3 mm above the umbilicus	Vaginal delivery: *n* = 23; cesarean section: *n* = 15/primiparous with a singleton/10 to 12 weeks pp	Measurements at rest and during abdominal crunch, drawing-in, and drawing-in + abdominal crunch exercises both for the vaginal delivery group and the cesarean section group	Single test	Single test	Not reported	IRD/ultrasound assessment at 2 cm above and below the umbilicus	Above the umbilicus: rest vs abdominal crunch: significant reduction in IRD (MD: 4.2 mm; 95% CI 0.5−7.9; *p*=0.02). Below the umbilicus: rest vs ADIM: significant increase in IRD (MD: −4.2 mm; 95% CI −7.9 to −0.5; *p*=0.02)
Mota et al. [51]	Longitudinal descriptive exploratory study/ultrasound assessment during tasks	*N*=84 pregnant and postpartum women/age: 32.1 (25−37)/DRA cut-off points not reported, IRD at 6 weeks pp: 26.8±9.3 mm at the umbilicus	Vaginal delivery (61.9%) and cesarean section (38.1%)/primiparous women/assessment points at gestational weeks 35−41, and at postpartum weeks 6−8, 12−14, and 24−26	Measurements at rest and during abdominal crunch, and drawing-in maneuver	Non-applicable	Four testing sessions (one session during pregnancy between gestational weeks 35 and 41, and three sessions postpartum (6−8 weeks postpartum, 12−14 weeks postpartum, and 24−26 weeks postpartum))	No drop-outs/all (*n*=84 participants) completed the assessment	IRD/ultrasound assessment 2 cm and 5 cm above the umbilicus and 2 cm below the umbilicus	In-drawing maneuver: gestational weeks 35−41: significant decrease in IRD with drawing-in 2 cm below the umbilicus (−3.8±11.8 mm). Postpartum period: significant increase in IRD with in-drawing below the umbilicus at postpartum weeks 6−8 (3.0±7.1 mm), 12−14 (1.8±5.6 mm), and 24−26 (2.5±5.2 mm). Abdominal crunch: gestational weeks 35−41: significant decrease in IRD at 2 cm below the umbilicus (−20.9±14.5 mm), at 2 cm above the umbilicus (−17.1±11.8 mm), and at 5 cm above the umbilicus (−11.2±12.5 mm). Postpartum period: significant decrease in IRD during postpartum weeks 6−8 and 12−14 at 2 cm below the umbilicus (−5.1±8.2 mm; −3.6±7.0 mm), at 2 cm above the umbilicus (−5.6±8.1 mm; −2.8±4.8 mm), and at 5 cm above the umbilicus (−4.4±6.6 mm; −1.6±4.6 mm). Significant decrease in IRD during postpartum weeks 24−26 at 2 cm above the umbilicus (−2.5±5.2 mm) and 5 cm above the umbilicus (−2.1±4.6 mm)
Chiarello et al. [30]	Cross-sectional study/ultrasound assessment during tasks	*N*=56 subjects (male, 11; nulliparous female, 22; parous female, 23)/age 18−65/DRA (IRD ≥2 cm) in 14 subjects (4 men and 10 parous women), mean IRD: 1.62±1.04 cm, nulliparous women IRD: 0.75±0.43 cm, parous women IRD: 2.03±1.05 cm at rest	Parity and delivery for parous women not reported/mean time since last delivery: 8.2 years	Measurements at rest and during active contraction (curl-up)	Single test	Single test	Not reported	IRD/ultrasound assessment at 4.5 cm above and below the midpoint of the umbilicus	Above the umbilicus: significant decrease in IRD for parous women from rest to curl-up (*p*=0.04, partial η^2^=0.179). Below the umbilicus: significant decrease in IRD for parous women from rest to curl-up (*p*=0.012, partial η^2^=0.256)
Lee and Hodges [12]	Cross-sectional study/ultrasound assessment during tasks	*N*=41 subjects, women with DRA, *n*=26; healthy controls, *n*= 17 (11 nulliparous women, 6 men)/age: DRA women 34±4, female controls 25±2, male controls 28±3/DRA >22 mm at 30 mm above the umbilicus, or >15 mm inferior to the xiphoid	Delivery mode not reported/1 nulliparous, 25 parous women/time since delivery not reported	Measurements at rest and during automatic curl-up, TrA preactivation, curl-up with TrA preactivation	Single test	Single test	Not reported	IRD, DI/ultrasound assessment at 3 cm above the umbilicus (U-point) and below the xiphoid (UX-point)	Automatic curl-up: significant decrease in IRD in DRA participants (*p*<0.001) TrA + curl-up: •IRD reduced at the U-point (*p*<0.001) but less than the reduction during an automatic curl-up in DRA participants. •IRD increased at U-point (*p*=0.02) and UX-point (*p*=0.007) for the control group. •DI increased from rest to automatic curl-up (*p*<0.05) but did not change during the TrA curl-up (*p*≥0.43). DI was greater during automatic curl-up than TrA curl-up (*p*=0.01)
Theodorsen et al. [31]	Cross-sectional study/ultrasound assessment during tasks	*p*=38/age: 34.6±4.0/postpartum women with DRA (at least two finger widths or more at the level of the umbilicus, and/or 2 cm below and above the umbilicus, women with an abdominal wall protrusion), IRD at rest: 2 cm above the umbilicus 25.7±8.5 mm, 2 cm below the umbilicus 21.0±7.9 mm	Vaginal delivery and cesarean section/primiparous and multiparous, single and multiple births/<6 months after delivery	Measurements at rest, during PFM contraction, during TrAM contraction, and combined PFM and TrAM contraction	Single test	Single test	Not reported	IRD/ultrasound assessment at 2 cm above and 2 cm below the umbilicus	Above the umbilicus: •significant increase in IRD during PFM vs rest: (MD: 1.2 [95% CI 0.7−1.7) mm, *p* < 0.001]) and during TrAM vs rest (MD: 2.8 [95% CI 1.9−3.6] mm, *p*<0.001). •Significant increase in IRD during PFM + TrAM vs rest (MD: 3.9 [95% CI 2.8−5.0] mm, *p*<0.001). •Significant increase in IRD during TrAM vs PFM (MD: 1.6 mm [95% CI 1.0− 2.1], *p*<0.001). Below the umbilicus: •Significant increase in IRD during PFM vs rest (MD: 0.9 [95% CI 0.4−1.6] mm, *p*<0.002) and during TrAM vs rest (MD: 2.3 [95% CI 1.5−3.1] mm, *p*<0.001). •Significant increase in IRD during PFM + TrAM vs rest (MD 3.3 [95% CI 2.4− 4.2] mm, *p*<0.001). •Significant increase in IRD during TrAM vs PFM (MD: 1.3 mm [95% CI 0.8−1.8], *p*<0.001)
Gluppe et al. [36]	Cross-sectional study/ultrasound assessment during tasks	*N*=38/age: 36.2±5.2/DRA diagnosis (IRD ˃25 mm, 2 cm above or 2 cm below the umbilicus) or observable protrusion during a curl-up, rest IRD at 2 cm above the umbilicus 43.6±12.7 mm, at 2 cm below the umbilicus 32.9±13.1 mm	Vaginal delivery and cesarean section/primiparous and multiparous/>6 weeks pp	Measurements at rest, head-lift, curl-up, PFM contraction, PFM contraction + curl-up, maximal in-drawing, PFM contraction + maximal in-drawing, pelvic tilt, and twisted curl-up	Single test	Single test	Not reported	IRD/ultrasound measurements at 2 cm above and 2 cm below the umbilicus	IRD decreased at 2 cm above the umbilicus during: rest vs head lift (*p*<0.01,d=1.06). Rest vs curl-up (*p*˂ 0.01, d=0.77). Rest vs PFM contraction + curl-up (*p*<0.01, d=0.49). Rest vs twisted curl-up (*p*˂0.01, d= 0.99). IRD decreased at 2 cm below the umbilicus during: rest vs head lift (*p*˂ 0.01, d= 0.71). Rest vs twisted curl-up (*p*=0.02, d=0.40). IRD increased at 2 cm below the umbilicus during: rest vs PFM contraction (*p*=0.02, d=0.41). Rest vs maximal in-drawing (*p*˂0.01, d= 0.60). Rest vs PFM contraction + maximal in-drawing (*p*˂0.01, d=0.61)
Leopold et al. [32]	Prospective cohort observational study/effectiveness of intervention on IRD, disability, continence, pelvic floor function, body image	Initial phase *N*=43 postpartum women with DRA, maintenance phase: *N*=19 postpartum women with DRA/age: 36.7±3.5/IRD ≥2 cm, rest IRD above the umbilicus 3.5±1.1 cm, rest IRD below the umbilicus 2.2±0.8 cm	Cesarean section and vaginal delivery/primiparous and multiparous/12 weeks to 3 years pp	Online core strengthening program: daily transversus abdominis activation with coordinated breathing and pelvic floor muscle engagement, coaching on alignment, posture, and proper core engagement, education on how to optimally engage the core muscles, specific low-impact weekly workouts (aerobic and resistance exercises)/no comparison	Transversus abdominis activation with coordinated breathing and pelvic floor muscle engagement: 10 min daily, low-impact weekly workouts: 20−30 min, up to 4× per week/daily progression of exercises/initial phase: 12 weeks’ duration, maintenance phase (*N*=19) 12 weeks’ duration	At baseline and at 12 weeks (end of intervention)/follow-up (maintenance phase *N*=19) at 24 weeks	Exercise program participants, *n*=56; drop-outs: initial phase: 13, maintenance phase: no drop-outs/all participants (*n*=43) completed the exercise program, participant compliance: 72%	IRD, LBP-related disability score (QBPDS), stress urinary incontinence score (ICIQ-UI), pelvic floor function score (PISQ-12), body image score (BSQ-16A)/ultrasound assessment at 3 cm above and below the umbilicus	Initial phase: IRD at rest decreased above (*p*<0.001) and below the umbilicus (*p*=0.009), IRD during contraction decreased above (*p*=0.037) and below the umbilicus (*p*<0.001), QBPDS decreased (*p*=0.002) and ICIQ-UI decreased (*p*=0.001) significantly. Maintenance phase: IRD at rest decreased above (*p*=0.007) and below the umbilicus (*p*<0.001), IRD during contraction decreased below the umbilicus (*p*<0.001)
Depledge et al. [41]	Cross-sectional study/ultrasound assessment during tasks	*N*=32 parous women/age: 32±4.6/IRD >2-finger breadth (mean IRD above the umbilicus 3.5±1.1 cm, mean IRD below the umbilicus: 2.6±1.2 cm)	Vaginal delivery/primi- and multiparous/2 weeks to 4 weeks pp	Measurements during rest; crook-lying abdominal “drawing-in” exercise; crook-lying trunk curl-up; early Sahrmann level leg raise; McGill side-lying plank. The curl-up and abdominal “drawing in” exercises were assessed a) wearing Tubigrip, b) with taping across the diastasis	Single test	Single test	Drop-outs: *n*=3/all other participants (*n*=29) completed the assessment	IRD/measured by ultrasound at 2 cm above and 2 cm below the umbilicus	Curl-up vs rest: significant decrease of IRD (19%, *p*<0.05) at both sites. No significant difference between rest and other exercises. Tubigrip vs taping vs no support: significant reduction of IRD (7%, *p*<0.05) with Tubigrip at rest. No significant effect (*p*>0.05) across supports during curl-up and drawing-in at both sites
Arranz-Martín et al. [22]	Short-term cross-sectional study/ultrasound assessment during tasks	*N*=46/age: 34.6±4/postpartum women (baseline IRD not reported), postpartum women (DRA cut-off points not reported), IRD at 2 cm above the umbilicus during rest: 2.31 cm (2.09−2.53)	Vaginal delivery/primiparous, singleton/3 months pp	Exercise program before testing (7th to 14th week postpartum): PFM and abdominal muscle exercises, ADIM, AHE. Testing: measurements during rest, during an AHE performed in a supine position, during a SCU, during an ADIM, and a SCU/comparisons between maneuvers	Exercise program before testing: supervised sessions: 10−33 AHEs, 5−10 sets of PFM contractions, 5−10 sets of TrA contractions (ADIM), 45 min sessions, 2× per week. At-home sessions: at least 3 AHEs, PFMs, and TrA contractions, 2× per week/progression not reported/8 weeks total (16 sessions)	Single test	Not reported	RA and lateral abdominal muscle thickness, IRD, LA distortion/ultrasound measurements at I-point: 2 cm below the umbilicus, S-point: 2 cm above the umbilicus, X-point: mid-point between the umbilicus and xiphoid process	IRD decreased significantly during: SCU at X- (0.34 cm, *p*=0.38) and S-point (0.49, *p*< 0.001). No significant differences between IRD at rest and AHE, ADIM, or ADIM+SCU exercise/increased occurrence of distortion. •SCU-rest at S- and I-point (41.3%, *p*<0.001). • ADIM+SCU–AHE at S-point (21.7%, *p*=0.041). Decreased occurrence of distortion ×ADIM+SCU-SCU at I (28.3%, *p*=0.023) and S point (28.3%%, *p*=0.045)
Djivoh and De Jaeger [38]	Cross-sectional study/IRD assessment during tasks using a caliper	*N*=42 parous women/age 28−54/IRD >15 mm at 20 mm above the umbilicus during a head-lift, mean IRD at 20 mm above the umbilicus during a head-lift 20.3±3.9 mm	Delivery mode not reported/primi- and multi-parous/7 months to >10 years pp	Measurements during head-lift, curl-up, sit-up, drawing-in + curl-up	Single test	Single test	Drop-outs: *n*=23/all other participants (*n*=19) completed the assessment	IRD/measured by caliper at 20 mm above the umbilicus	Significant decrease in IRD during curl-up 8.1±2.6 mm (*p*<0.001) and sit-up 8.2±3.0 mm (*p*<0.001) compared with head-lift. No significant decrease in IRD during drawing-in + curl-up compared with head-lift. No significant decrease in IRD during sit-ups compared with curl-up

*IRD* inter-recti distance, *DRA* diastasis recti abdominis, *pp* postpartum, *PFM* pelvic floor muscle, *HEP* home exercise program, *ODI* Oswestry Disability Index, *PFDI*, Pelvic Floor Disability Index, *NMES* neuromuscular electrical stimulation, *X-U/2* halfway between the umbilicus and the xiphoid process, *U-P/2* halfway between the umbilicus and the pubic symphysis, *TrA(M)* transversus abdominis (muscle), *RDQ* Roland−Morris Disability Questionnaire, *PF-10* Physical Functioning scale, *MD* mean difference, *MBSRQ* Multidimensional Body-Self Relations Questionnaire, *IFSAC* inventory of functional status after childbirth, *UHBE* Unilateral Hip Bridge Endurance Test, *RA* rectus abdominis, *MAPP-QOL* Maternal Quality Of Life Questionnaire, *EO* external oblique, *IO* internal oblique, *BAPFMT* biofeedback-assisted pelvic floor muscle training, *SF-36* Short-Form Health Survey-36, *PCS* physical components summary, *MCS* mental components summary, *VAS* Visual Analogue Scale, *STS* suspension training system, *ISoM−ISoT* isometric−isotonic core stabilization exercises, *ADIM* abdominal drawing-in maneuver, *DI* distortion index, *U* umbilicus, *LBP* low-back pain, *QBPDS* Quebec Back Pain Disability Scale, *ICIQ-UI* International Consultation on Incontinence Questionnaire-Urinary Incontinence, *PISQ-12* Pelvic Organ Prolapse/Urinary Incontinence Sexual Questionnaire, *BSQ-16A* Body Shape Questionnaire, *AHE* abdominal hypopressive exercise, *SCU* semi curl-up, *LA* linea alba

## Abbreviations

ADIM: Abdominal drawing-in maneuver
AHE(s): Abdominal hypopressive exercise(s)
BAPFMT: Biofeedback-assisted pelvic floor muscle training
DRA: Diastasis recti abdominis
IRD: Inter-recti distance
LA: Linea alba
NMES: Neuromuscular electrical stimulation
PFM(s): Pelvic floor muscle(s)
QoL: Quality of life
RA: Rectus abdominis
STS: Suspension training system
TrA: Transversus abdominis
