# Supplementary material for: Diastasis Recti Abdominis Rehabilitation in the Postpartum Period: A Scoping Review of Current Clinical Practice
Source: Int Urogynecol J. 2024 Feb 10;35(3):491–520. doi: 10.1007/s00192-024-05727-1 (PMC11023973; doi:10.1007/s00192-024-05727-1)
Supplement: Supplementary file 1 — (DOCX 10563 kb) [file 192_2024_5727_MOESM1_ESM.docx]

***Electronic Supplementary Material***


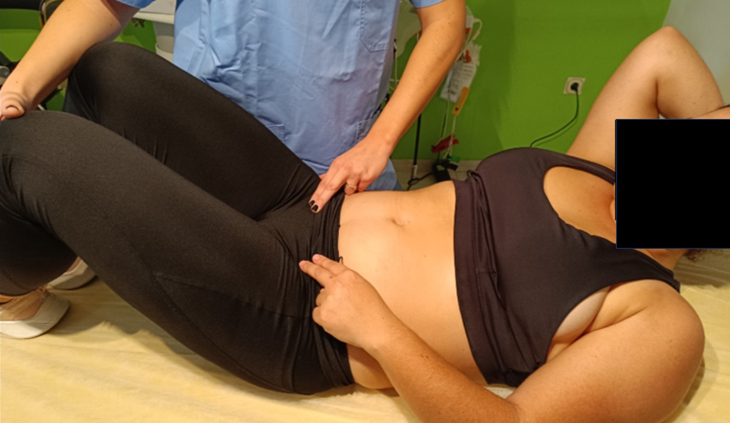


**Illustration 1.** **Co-contraction of transversus abdominis (TrA) and rectus abdominis muscles.** In this progression of TrA activation exercise, the patient elevates the head and upper torso while maintaining an abdominal drawing-in maneuver (ADIM). The patient is positioned in supine with legs bent and arms supporting the head. They are guided to take a deep breath keeping the abdomen relaxed and then to fully exhale slowly. At the end of expiration, the patient is asked to pull the belly button towards the spine and upwards towards the thorax and to elevate the head and upper torso as if to perform a crunch, while maintaining the contraction. The physiotherapist and the patient can palpate TrA muscle activation just medially of the anterior superior iliac spine, using two fingers. Adequate control of the deep system is achieved when there is no visible abdominal bulge or invagination during trunk flexion.


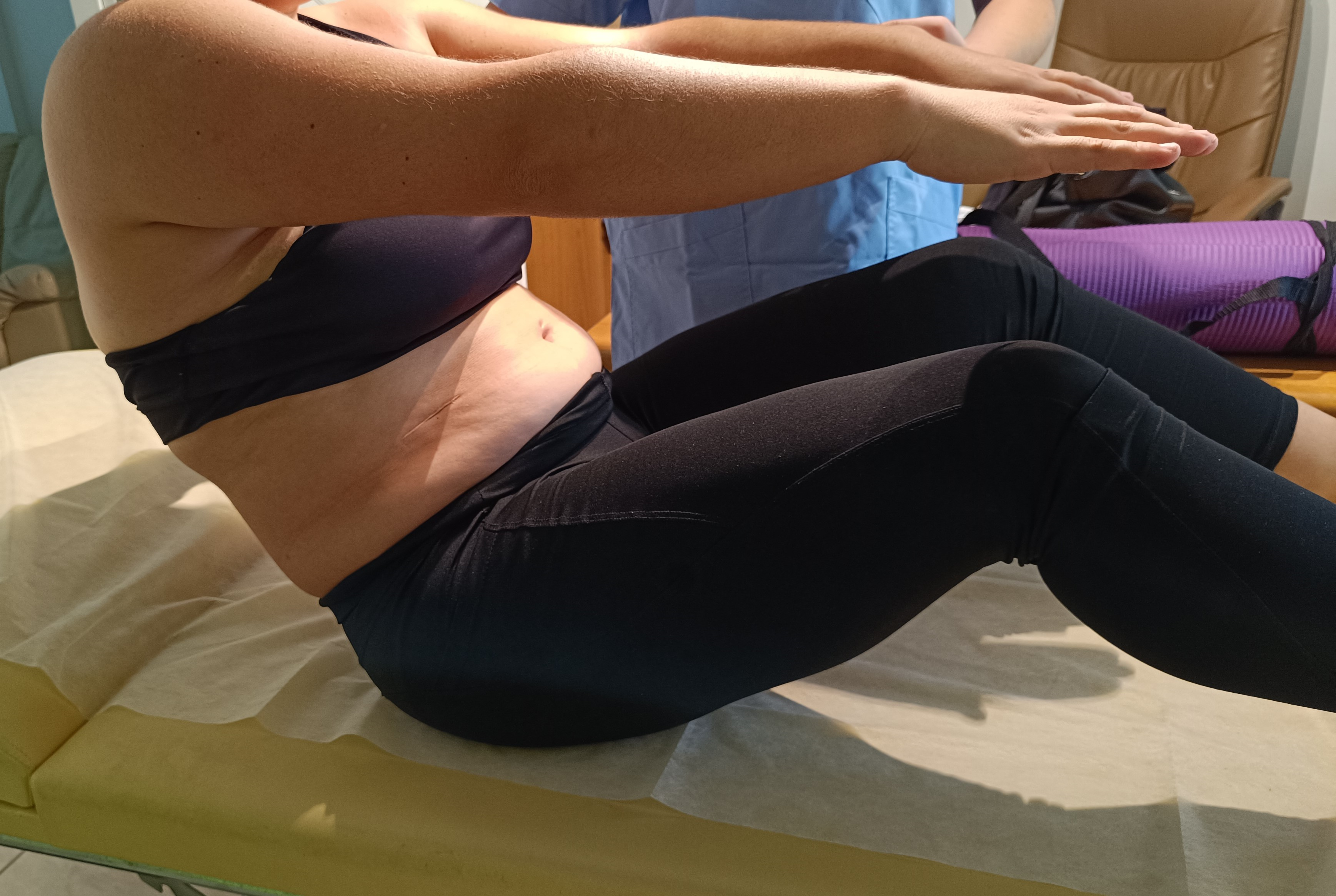


**Illustration 2. Eccentric activation of the abdominal muscles.** The patient, seated upright with the trunk extended and knees bent, places feet on the examination bed. Following instructions, the patient gradually rolls backward, maintaining a flexed trunk, and lowers in a controlled manner while engaging the abdominal muscles. Upon reaching a position where both hands are above the knees, the patient returns to an upright seating position. This exercise might be combined with an abdominal drawing-in maneuver (ADIM) and/or pelvic floor muscle (PFM) contraction. Eccentric contraction involves muscle lengthening under tension, as opposed to concentric contraction, where the muscle shortens during contraction. This exercise incorporates an eccentric phase during the backward roll and a concentric phase when the patient bends their trunk forward to return to an upright position. To isolate the eccentric contraction, the physiotherapist may assist the patient during the return or guide them to use their arms to push on the bed for assistance.


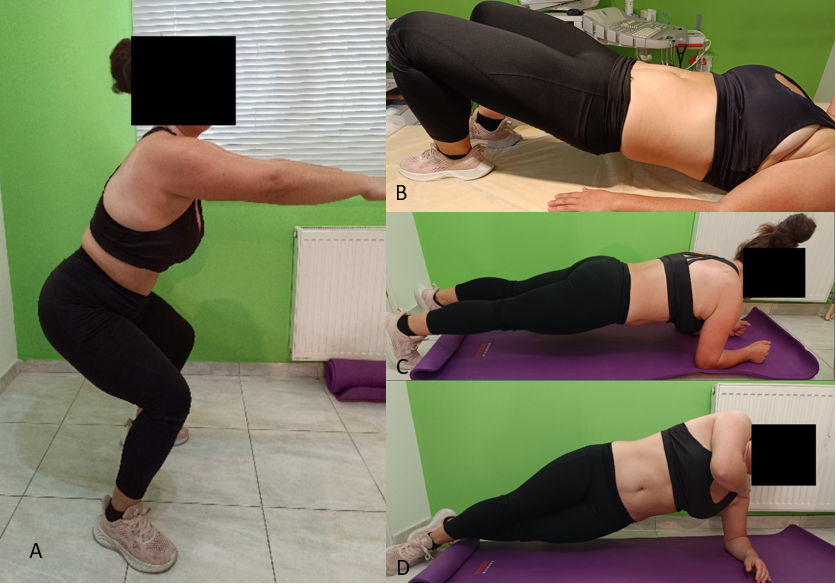


**Illustration 3. Functional exercises.** These exercises are designed to engage multiple muscle groups simultaneously, requiring effective inner unit control. All four exercises are enhanced by combining an abdominal drawing-in maneuver (ADIM) and/or pelvic floor muscle (PFM) contraction. **A.** Squat exercise. The patient stands upright with legs hip-width apart, bending the knees and hips while driving both hips backward as if sitting on a chair. Arms can extend forward to maintain balance. Lowering of the hips is performed until the inner unit contraction can be maintained, followed by a return to the initial position. **B.** Bridge exercise. In a supine position with legs bent, hips apart, and feet flat on the bed, the patient maintains an inner unit contraction while activating hip extensors, pushing to elevate the pelvis. The movement continues until the point where the contraction can be maintained, followed by a return to the initial position. **C.** Front plank exercise. Starting in a 4-point kneeling position on elbows and knees, the patient activates the inner unit and extends their legs backward to form a straight line with hips and trunk at the same height. The patient maintains the position as long as they can sustain both the inner unit contraction and the instructed form. **D.** Side plank exercise. In a side-lying position with the trunk supported on the elbow, the patient elevates the pelvis and extends the legs, forming a straight line with their body. The upper leg is positioned in front of the other. The patient maintains the position for as long as they can sustain both the inner unit contraction and the instructed form.
